# Dual-function transaminases with hybrid nanoflower for the production of value-added chemicals from biobased levulinic acid

**DOI:** 10.3389/fbioe.2023.1280464

**Published:** 2023-11-16

**Authors:** Taresh P. Khobragade, Pritam Giri, Amol D. Pagar, Mahesh D. Patil, Sharad Sarak, Sangwoo Joo, Younghwan Goh, Seohee Jung, Hyunseok Yoon, Subin Yun, Youkyoung Kwon, Hyungdon Yun

**Affiliations:** ^1^ Department of Systems Biotechnology, Konkuk University, Seoul, Republic of Korea; ^2^ Department of Nanomaterials and Application Technology, Center of Innovative and Applied Bioprocessing (CIAB), Mohali, Punjab, India

**Keywords:** biomass, amino donor, transaminase, nanoflower, fusion protein

## Abstract

The U.S. Department of Energy has listed levulinic acid (LA) as one of the top 12 compounds derived from biomass. LA has gained much attention owing to its conversion into enantiopure 4-aminopentanoic acid through an amination reaction. Herein, we developed a coupled-enzyme recyclable cascade employing two transaminases (TAs) for the synthesis of (*S*)-4-aminopentanoic acid. TAs were first utilized to convert LA into (*S*)-4-aminopentanoic acid using (*S*)-α-Methylbenzylamine [(*S*)-α-MBA] as an amino donor. The deaminated (*S*)-α-MBA i.e., acetophenone was recycled back using a second TAs while using isopropyl amine (IPA) amino donor to generate easily removable acetone. Enzymatic reactions were carried out using different systems, with conversions ranging from 30% to 80%. Furthermore, the hybrid nanoflowers (HNF) of the fusion protein were constructed which afforded complete biocatalytic conversion of LA to the desired (*S*)-4-aminopentanoic acid. The created HNF demonstrated storage stability for over a month and can be reused for up to 7 sequential cycles. A preparative scale reaction (100 mL) achieved the complete conversion with an isolated yield of 62%. Furthermore, the applicability of this recycling system was tested with different β-keto ester substrates, wherein 18%–48% of corresponding β-amino acids were synthesized. Finally, this recycling system was applied for the biosynthesis of pharmaceutical important drug sitagliptin intermediate ((*R*)-3-amino-4-(2,4,5-triflurophenyl) butanoic acid) with an excellent conversion 82%.

## 1 Introduction

Biomass has gained increasing attention for the past few decades because of the unique renewable carbonaceous resource on the earth and is regarded as an alternative resource to fossil fuels (Changzhi et al., 2015; Mahlet et al., 2020; Yoo et al., 2023). Moreover, biomass can serve as an exclusive source of carbon-based fuels, specialty chemicals and value-added chemicals for the Green and sustainable future (Akwasi et al., 2021; Gupta et al., 2022). Amination of biomass is an indispensable process that plays a pivotal role in the production of a diverse array of fine chemicals, pharmaceuticals, and agricultural products (Froidevaux et al., 2016). Traditional organo-catalytic or chemical processes, however, frequently call for pricey starting materials and/or transition-metal catalysts, laborious protection and deprotection stages, and high pressures and/or temperatures (Collet et al., 2009). Alternatively, biocatalytic processes are advantageous such as simpler, safer for the environment, more atom-efficient, and highly enantioselective along with some disadvantage like stability which include inactivation at high temperature, solvents, pH value, ionic strength, and salt type (Bommarius, 2015; Lin and Tao, 2017; Patil et al., 2018; Giri et al., 2021; Chook et al., 2023; Lewis et al., 2023).

Recent years have evidenced a significantly increased interest in the enzymatic synthesis of value-added chemicals from biomass via amination routes. Enzymes such as transaminases (TAs), monoamine oxidases, amine dehydrogenases, phenylalanine ammonia lyases, imine reductases, lipases, P450 monooxygenases, Pictet-Spenglerases, and berberine bridge enzymes constitute a proficient pool of biocatalysts for the synthesis of various amines (Patil et al., 2018b; Murshidul et al., 2018; Giri et al., 2021). In particular, biocatalytic transaminations are crucial for the synthesis of nylon monomers, diamines, and furan-based compounds (Silvianti et al., 2020; Zhu and Yin, 2021). In comparison to other enzymes, TAs offer numerous advantages including high catalytic activity and stereoselectivity, broad substrate specificity, and no need for an additional cofactor. Likewise, TAs can kinetically resolve racemic amines and produce optically pure amines from the necessary prochiral carbonyl compounds (Hohne and Bornscheuer, 2009; Mathew and Yun, 2012; Guo and Berglund, 2017; Patil et al., 2018a). For instance, a higher concentration of nylon monomer was synthesized from renewable fatty acids utilizing a multi-enzyme cascade employing a TA (Yoo et al., 2022). In another study, an *E coli*-based modularization strategy was applied for the synthesis of 1,6-hexamethylenediamine and associated α, ω-diamines from corresponding cycloalkanols (Sarak et al., 2023). Lee et al. reported a multi-step biotransformation reaction to produce long chain aliphatic amines from renewable fatty acids (Cha et al., 2020). A wild-type amine dehydrogenase that may reductively ameliorate biobased LA to (*S*)-4-aminopentanoic acid was recently discovered by Mayol et al., 2016. He’s group reported a whole-cell reaction by employing the transaminase from *Chromobacterium violaceum* for the valorization of biomass-derived 5-Hydroxymethylfurfural to 5-Hydroxymethylfurfural amine (Wang et al., 2022).

Despite being potential biocatalysts, the industrial applications of TAs are often confined by limited operation stability in harsh reaction conditions such as higher temperatures, acidic or basic pH, and the presence of organic solvents (Patil et al., 2018). Nano-biocatalysis primarily concerned with immobilization of enzyme on nanoscale supports using traditional immobilization techniques such as physical adsorption, covalent binding, entrapment, and carrier free immobilization. Hybrid organic–inorganic nano-formulations (NFs) show improved enzymatic activity and stability compared to free enzymes which may be attributed to the confinement of the enzyme in the core of the NFs and high-surface-area (Lv et al., 2020). The combined functionalities of the protein and the inorganic material of the hybrid NFs enable its application in biosensors, biofuel cells, and biocatalysis (Li et al., 2020).

**FIGURE 1:**
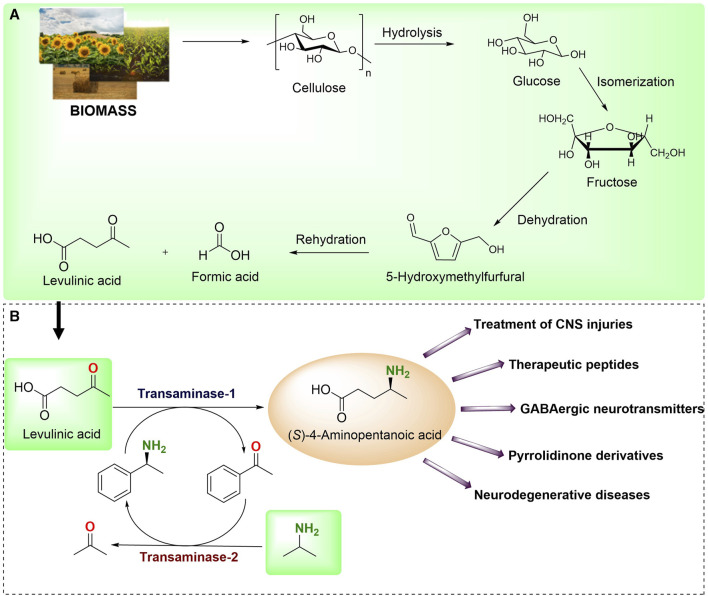
**(A)** Chemical synthesis of LA from biomass source. **(B)** Enzymatic synthesis of (*S*)-4-aminopentanoic acid from biobased LA reported in the present study and its application.

LA has been listed by the U.S. Department of Energy as one of the top 12 compounds derived from biomass (Figure 1A) (Kang et al., 2018). LA has gained more attention because of its plausible conversion into chiral 4-aminopentanoic acid through an amination reaction (Liu et al., 2022). The optically pure (*S*)-4-aminopentanoic acid serves as a pivotal precursor in the synthesis of therapeutic molecules, pyrrolidinone derivatives and is used as false GABAergic neurotransmitters (Cai et al., 2020; Wawro et al., 2021; Zhou et al., 2022). Generally, the chemical conversion of LA into chiral 4-aminopentanoic acid still suffers from poor stereoselectivity (Wu et al., 2019). Recent years have witnessed the development of various biocatalytic approaches to circumvent poor stereoselectivity (Gupta et al., 2022). Recently, Jiang et al. discovered an (*S*)-TA that showed distinct activity toward LA (10 mM) with 66% conversion in a 1 mL reaction (Jiang et al., 2014). In another study, Mayol et al., 2016. transformed the 500 mM LA to (*S*)-4-aminopentanoic acid employing a wild-type amine dehydrogenase with 88% conversion in a 9 mL reaction. After that, Cai et al. used directed evolution to create an active mutant of amine dehydrogenase with increased activity, resulting in a successful synthesis of (*S*)-4-aminopentanoic acid by employing 500 mM LA as a substrate with 97% conversion in a 200 mL reaction (Cai et al., 2020). However, amine dehydrogenases use excess ammonia, which poses difficulty in the isolation of final products ((*S*)-4-aminopentanoic acid) which included the extra steps and instrument. Moreover, additional enzymes are required for the recycling of costly cofactors.

We envisaged developing a biocatalytic cascade for the synthesis of (*S*)-4-aminopentanoic acid from biobased LA. In this coupled-enzyme reaction, transaminase was utilized to transform LA into (*S*)-4-aminopentanoic acid using (*S*)-α-MBA as an amino donor. The deaminated (*S*)-α-MBA i.e., acetophenone was recycled back using another transaminase while using isopropyl amine (IPA) as amino donor to generate easily removable acetone (Figure 1B). To begin with, we examined a panel of 30 in-house TAs and evaluated their selectivity towards IPA and (*S*)-α-MBA. After choosing the appropriate TAs, we investigate the effectiveness of single- and two-cell systems for better conversions of LA to (*S*)-4-aminopentanoic acid. In addition, we synthesized the nanoflowers of TA and subsequently employed them in the enzymatic reactions resulting in excellent conversions. Furthermore, the preparative scale reaction was performed with the isolation of the final product. The applicability of this system was tested with different β-keto ester substrates.

## 2 Materials and methods

### 2.1 Chemicals and enzymes

LA acids, β-keto esters, β-amino acids, *Candida rugosa* lipase (Catalog No. L1754), (S)-α-methylbenzylamine [(*S*)-α-MBA], 2,3,4,6-Tetra-O-acetyl-β-D-glucopyranosyl isothiocyanate (GITC), alanine, ammonium formate, pyridoxal 5′-phosphate hydrate (PLP), perchloric acid (PCA), dimethyl sulfoxide (DMSO) were purchased from Sigma-Aldrich (Sigma-Aldrich Korea, Seoul, South Korea) and Diethyl ethoxymethylenemalonate (DEEMM) was purchased from Tokyo chemical industry Co., Ltd. Ethyl 3-oxo-4-(2,4,5-trifluorophenyl) butanoate were obtained from CKD BiO Corp, Korea. The genes encoding various TAs (except TAPO and TAST), FDH, FDHm and AlaDH were synthesized by Bionics (Seoul, Korea).

### 2.2 The expression and purification of enzymes

The genes that code for the diverse TAs were made by Bionics company (Seoul, Korea). The IPTG-inducible pET24ma vectors carrying each gene were then transformed into *E. coli* (BL21) cells and allowed to grow in 0.5 L of LB with kanamycin (50 mg/mL) at 37°C. When the OD_600_ reached 0.6–0.8, 0.1 mM IPTG was added to induce the cells following the overnight induction at 20°C. Next, centrifugation at 5,000 rpm for 10 min was used to harvest the *E. coli* (BL21) cells, which were washed twice in 200 mM Tris/HCl buffer (pH 7.0). A horn-type sonicator (Sonics& Material Inc., United States) was used to provide ultrasonic disruption to the washed cells after resuspending them in a 10 mL lysis buffer. To purify the C-terminally His_6_-tagged enzymes from the cell lysates, Ni-NTA agarose resin [Qiagen, Hilden, Germany] was used. The rising gradient of imidazole [50–250 mM] was used to elute the enzymes. An Amicon PM-10 ultrafiltration device was used to dialyze the eluted solution (protein) against a 20 mM Tris-HCl buffer (pH 7.4) to concentrate the protein purified.

### 2.3 Synthesis of hybrid nanoflower

The purified enzymes (TAs) (0.2 mg/mL) were dissolved in 10 mM phosphate buffer saline (pH 7.4), uniformly dispersed with 2 mM different salt solutions (Copper sulphate (CuSO_4_), potassium sulphate (K_2_SO_4_), sodium sulphate (Na_2_SO_4_), magnesium sulphate (MgSO_4_), zinc sulphate (ZnSO_4_), nickel sulphate (NiSO_4_), ferrous sulphate (FeSO_4_) and cobalt sulphate (CoSO_4_) and then incubated undisturbed at 4°C for 72 h. Organic−inorganic hybrid nanoflowers were obtained as a white precipitate at the bottom of the reaction vessel (Eppendorf). Synthesized nanoflowers were collected after centrifugation at 10000 *g* for 5 min and washed successively 2–3 times with 50 mM HEPES buffer (pH 6.0) to remove unbound protein and stored at 4°C for further use (Rai et al., 2018). Protein loading in the hybrid nanoflowers was determined using the Bradford assay. The synthesized hybrid nanoflowers were characterized by scanning electron microscopy (SEM).

### 2.4 Isolation of (*S*)-4-aminopentanoic acid from reaction mixture

A biocatalytic reaction was performed, in a total volume of 100 mL and after completion of the reaction with complete conversion, the reaction mixture was subjected to isolation and purification of the desired product. The reaction solution including the desired products was first centrifuged for 20 min, then the precipitate (HNF) was removed through filtration. Next, the supernatant layer pH was adjusted to pH 11 using 5 M NaOH, followed by extracted twice with ethyl acetate to get rid of the unreacted amine donor and other reaction intermediates that removed using organic solvents. After the separated aqueous layer was evaporated to reduce the volume, tert-butanol, sodium hydroxide (1.1 eq), and di-tert-butyl dicarbonate (Boc-anhydride 3.0 eq) were added in equal parts to begin the Boc-protection process. After being agitated at room temperature for 12 h, the reaction mixture was made more acidic (pH 5.0) by adding 6 M HCl. The resultant solution underwent two ethyl acetate extractions, a brine wash, a drying step over magnesium sulfate, and vacuum concentration. An equivalent volume of methanol and 4N HCl were then used to execute a Boc deprotection reaction for 1 h, and the resultant solution was then concentrated in a vacuum to produce the desired product with an isolated yield of 62%.

## 3 Results and discussion

### 3.1 Screening of transaminase for the amination of levulinic acid

**FIGURE 2:**
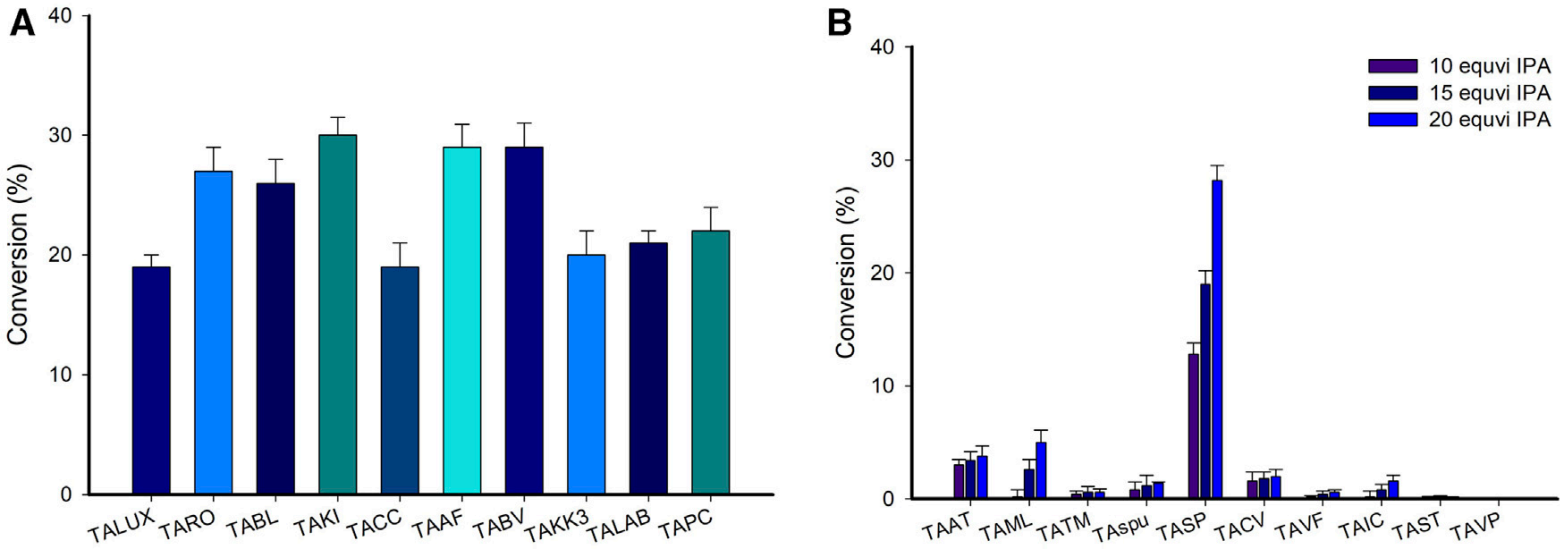
**(A)** A screening of various TAs for amination of LA employing (*S*)-α-MBA (Representative example of TAs, See Supplementary Figure S1). Reaction conditions: 10 mM LA as a substrate, 25 mM (*S*)-α-MBA, 0.1 mM PLP, and 100 mM Tris-HCl buffer pH 8.0 at 37°C for 24 h. **(B)** A screening of TAs for the conversion of acetophenone to (*S*)-MBA using isopropyl amine as an amino donor (Representative example of TAs, See Supplementary Figure S2). Reaction condition; 50 mM acetophenone substrate, IPA (10, 15, or 20 equivalent) 0.5 mM PLP, 12 mg_CDW_/ml cells of each TA, 37°C for 24 h.

The activity of TAs towards various substrates differs significantly, as it is largely controlled by steric constraints in the substrate-binding pockets of the enzyme (Jeon et al., 2022). For the TA-catalyzed reactions utilization of IPA as an amino donor is more favorable due to its cost effectiveness and easily removable coproduct acetone (Simon et al., 2014; Dawood et al., 2018). Moreover, to utilize IPA as an amino donor we screened the various TAs for the conversion of LA to its corresponding amine compound. However, there should not be any considerable conversion of LA using IPA as an amino donor (Supplementary Figure S1). Therefore, we further investigate TAs reactions using (*S*)-α-MBA as an amino donor for the synthesis of corresponding amine employing LA as a substrate. Generally, in TA-catalyzed reaction using (*S*)-α-MBA as an amino donor showed significant activity. A panel of 30 in-house TAs (Supplementary Table S1) was evaluated to find the best ones for accelerating the conversion of LA to the corresponding amine employing IPA and (*S*)-α-MBA as an amino donor. Following codon optimization, the genes encoding TAs from 30 distinct sources were synthesized, cloned in pET-24ma, and expressed in *E. coli* BL21 (DE3). For the initial screening, 200 μL reaction mixture containing LB medium, an antibiotic (kanamycin 50 mg/mL), IPTG, and whole-cells expressing individual enzymes was set up in a 96-well microtiter plate and incubated overnight at 37°C in an orbital shaker. The cells were harvested, and the LA amination reaction was carried out using them in a solution containing 10 mM LA as a substrate, 25 mM (*S*)-α-MBA or 100 mM IPA, 0.1 mM PLP, and 100 mM Tris-HCl buffer (pH 8.0) at 37°C for 24 h (Figure 2A, Supplementary Figure S2). The reaction was stopped with the addition of 10% PCA solution, and HPLC was used for analysis. While employing (*S*)-α-MBA as an amino donor, the majority of TAs were still able to successfully aminate the LA substrate, albeit at decreased conversion rates. The highest conversion rates were demonstrated by TAKI and TAAF (28%), followed by TAPI, TARO, and TABV with conversions ranging from 24% to 26%. Therefore, 5 TAs (TAKI, TAAF, TAPI, TARO, and TABV) were chosen for further investigation.

It is worth noting that transamination reactions catalyzed by TAs generally are thermodynamically limited (Voges et al., 2016). For instance, the generation of deaminated (*S*)-α-MBA, i.e., acetophenone after the amination of LA in the present study limits the progress of the forward reaction. The recycling or removal of the deaminated co-product can overcome the thermodynamic barrier of the reaction. We hypothesized that recycling acetophenone to (*S*)-α-MBA using IPA as an amino donor by another TA can circumvent this problem. Thus, the transamination efficiency of TAs was evaluated using acetophenone as a substrate while using IPA as an amino donor. We commenced our investigation to identify the optimal TA that can utilize IPA as an amino donor and generate (*S*)-MBA from acetophenone with high specificity. First, a whole-cell biotransformation using 50 mM acetophenone as the substrate, IPA at varying doses (corresponding to 10, 15, and 20 equivalent), for 24 h, was performed (Figure 2B, Supplementary Figure S3). Among the 30 investigated TAs, TASP demonstrated the highest conversion (30%) using 20 equivalent IPA as an amino donor. While two other TAs, *viz*. TAML and TAAT showed mediocre conversions of 6% and 4%, respectively, all the remaining TAs exhibited negligible conversions. The successful utilization of IPA by TASP corroborated the findings of the Kroutil and Bornscheuer group, who reported that a wide number of enzymes from the fold-type I group of amine transaminases (ATAs) use IPA while typically exhibiting (*S*)-selectivity (Simon et al., 2014; Dawood et al., 2018).

### 3.2 Coupled reaction employing two transaminases (TA_1_/TA_2_)

Asymmetric synthesis is one of the most practical and economically advantageous methods for producing an enantiopure target molecules since it can produce a theoretical yield of 100%. However, unfavorable reaction equilibrium is a crucial downside of this approach. Thus, equilibrium displacement approaches such as the use of excessive amounts of amine donor and/or rapid removal of co-products using enzymatic cascades have been developed (Green et al., 2014; Richter et al., 2015; Khobragade et al., 2021). Since preliminary experiments revealed the potential of TASP to recycle acetophenone, we attempted to establish a coupled enzyme reaction constituting both the transaminases in a single pot. The whole-cell biotransformation reaction was performed using five enzymes (TAKI, TAAF, TAPI, TARO and TABV) and coupled with TA2 (TASP) containing 10 mM LA as a substrate, 25 mM (*S*)-α-MBA, 100 mM IPA, 0.1 mM PLP, 6 mg_CDW_/mL of cell TA_1_ and 6 mg_CDW_/mL of cell TA_2_, 100 mM Tris-HCl buffer (pH 8.0), 37°C for 24 h (Supplementary Table S2). The highest conversion was observed by the combination of TASP with TAKI (58%), followed by TAAF (42%) and TABV (40%), and TARO (39%). In addition, the recycling of deaminated (*S*)-α-MBA and L-alanine generated by TA_1_ was also attempted using the TA recycling system and Alanine dehydrogenase (AlaDH) recycling system (Supplementary Figure S4). TA1/TASP was replaced by the TA_1_/AlaDH recycling system while using L-alanine as an amino donor. The recycling system employing two transaminases (TAKI/TASP) achieved the maximum conversion of 59%, whereas TAPI/TASP demonstrated the lowest conversion of 30%. On the other hand, the TA_1_/AlaDH system could achieve a mediocre conversion of 5%. Furthermore, no conversion was achieved with the use of IPA as an amino donor for TA_1_ enzymes (Supplementary Figure S5). These results demonstrated the suitability of the coupled-transaminase system (TA_1_/TA_2_) for the co-product removal and recycling of the amino donor.

The specific activities of previously reported TA (TABKV) (Jiang et al., 2014) and the best enzyme in the preliminary experiments (TAKI) (System A1) were determined to be 103 and 128 mU/mg, respectively. To compare the catalytic efficiency of the newly screened TAKI with the previously reported TABKV, the apparent kinetic parameters toward LA and the co-substrate (*S*)-α-MBA were investigated. (Supplementary Table S3). Marginally lower KM value (5.9 mM) in the case of TAKI led to a 3-fold increase in the catalytic efficiency compared to that by TABKV (Supplementary Table S3, entry 1). Moreover, an enzymatic reaction was performed where TABKV afforded 20% conversion of LA as a substrate and, the maximum conversion achieved by TAKI was 32% under identical reaction conditions (Supplementary Figure S6). Therefore, TAKI was used for further experiments.

**FIGURE 3:**
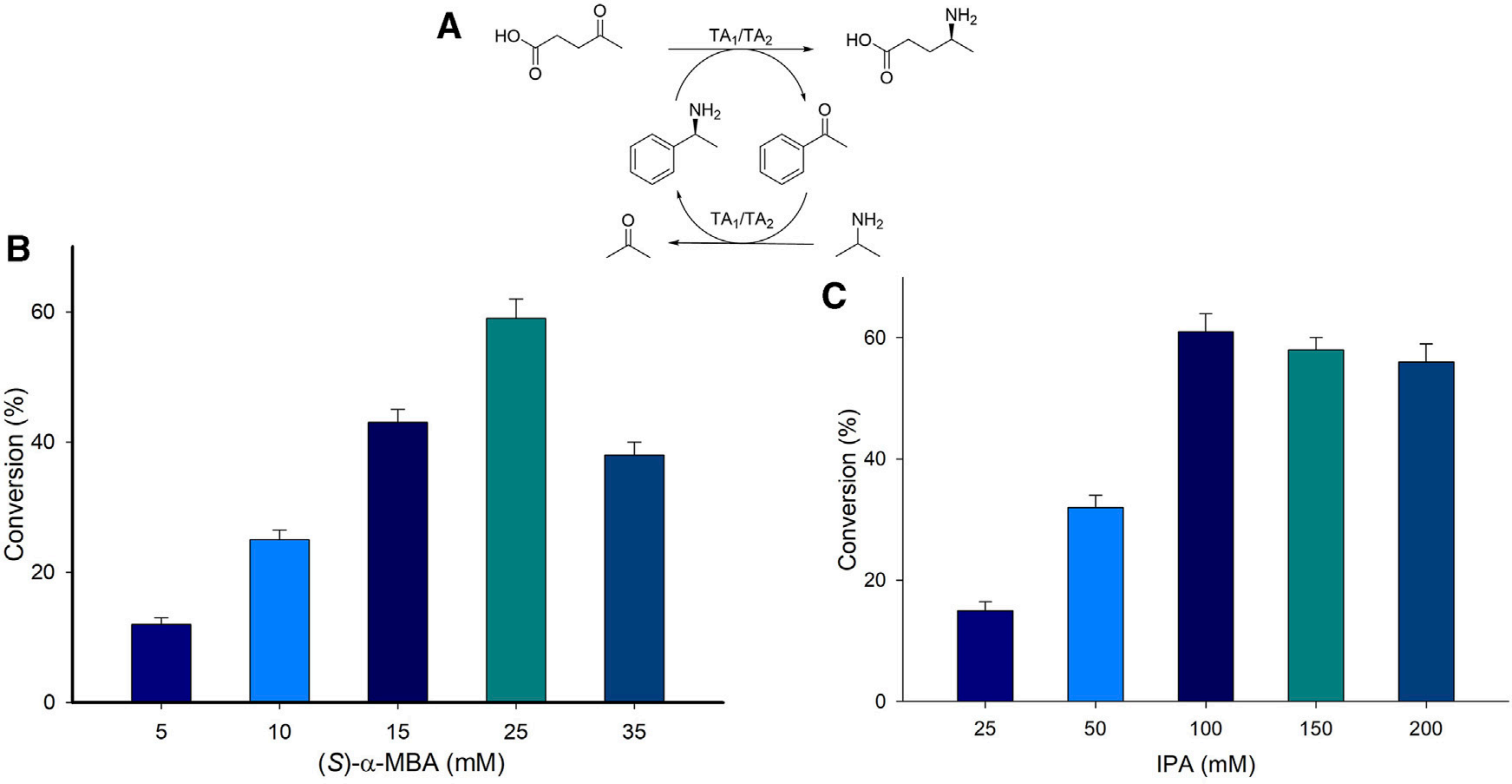
**(A)** Reaction scheme for the synthesis of (*S*)-4-aminopentanoic acid from biobased compound LA. **(B)** Amino donor optimization employing (*S*)-α-MBA. **(C)** Amino donor optimization employing IPA. Reaction condition; 10 mM LA substrate, 0.1 mM PLP, 6 mg_CDW_/mL of cell TAKI or 6 mg_CDW_/mL of cell TASP, 100 mM Tris-HCl buffer (pH 8.0), 37°C for 24 h.

The concentration of the amino donor in a biocatalytic transamination plays a pivotal role in driving the reaction equilibrium and ultimately in the product formation. A whole-cell reaction was carried out with varying concentrations of amino donor (*S*)-α-MBA ranging from 5 to 35 mM and 10 mM LA as a substrate with TAKI (Supplementary Figure S7). The highest conversion (30%) was displayed when 25 mM (*S*)-α-MBA was used, on the other hand only 4% conversion was afforded with the use of 5 mM (*S*)-α-MBA. An increase in the concentration of (*S*)-α-MBA beyond 25 mM exhibited a negative effect on the final product formation. Furthermore, a coupled transaminase reaction (TAKI/TASP) (System A2, Figure 3A) was performed with varying concentrations of amino donor (5–35 mM (*S*)-α-MBA), 10 mM LA, 100 mM IPA, 0.1 mM PLP, 6 mg_CDW_/mL of cell TA_1_ (TAKI) or 6 mg_CDW_/mL of cell TA_2_ (TASP), 100 mM Tris-HCl buffer (pH 8.0), 37°C for 24 h (Figure 3B). A linear increase in product formation was observed with the increasing concentration of (*S*)-α-MBA till 25 mM. The highest conversion of 60% was achieved with the use of 25 mM (*S*)-α-MBA. Further increase in the concentration of (*S*)-α-MBA beyond 25 mM showed a detrimental effect on the formation of (*S*)-4-aminopentanoic acid product from LA substrate. Therefore, 25 mM (*S*)-α-MBA was the most suitable for the reaction and was used in subsequent experiments.

Similarly, the effect of IPA concentration on the reaction was investigated by varying the concentration of IPA from 50 to 200 mM (Figure 3C). The highest conversion was achieved when 100 mM of IPA was used, whereas the lowest was for 50 mM IPA. The excess of IPA concentration of more than 10 equivalent led to lowered product formation. Hence, the IPA concentration was fixed at 100 mM for the following experiments.

### 3.3 Construction of single cell system by co-expression and creation of dual-function fusion protein

**FIGURE 4:**
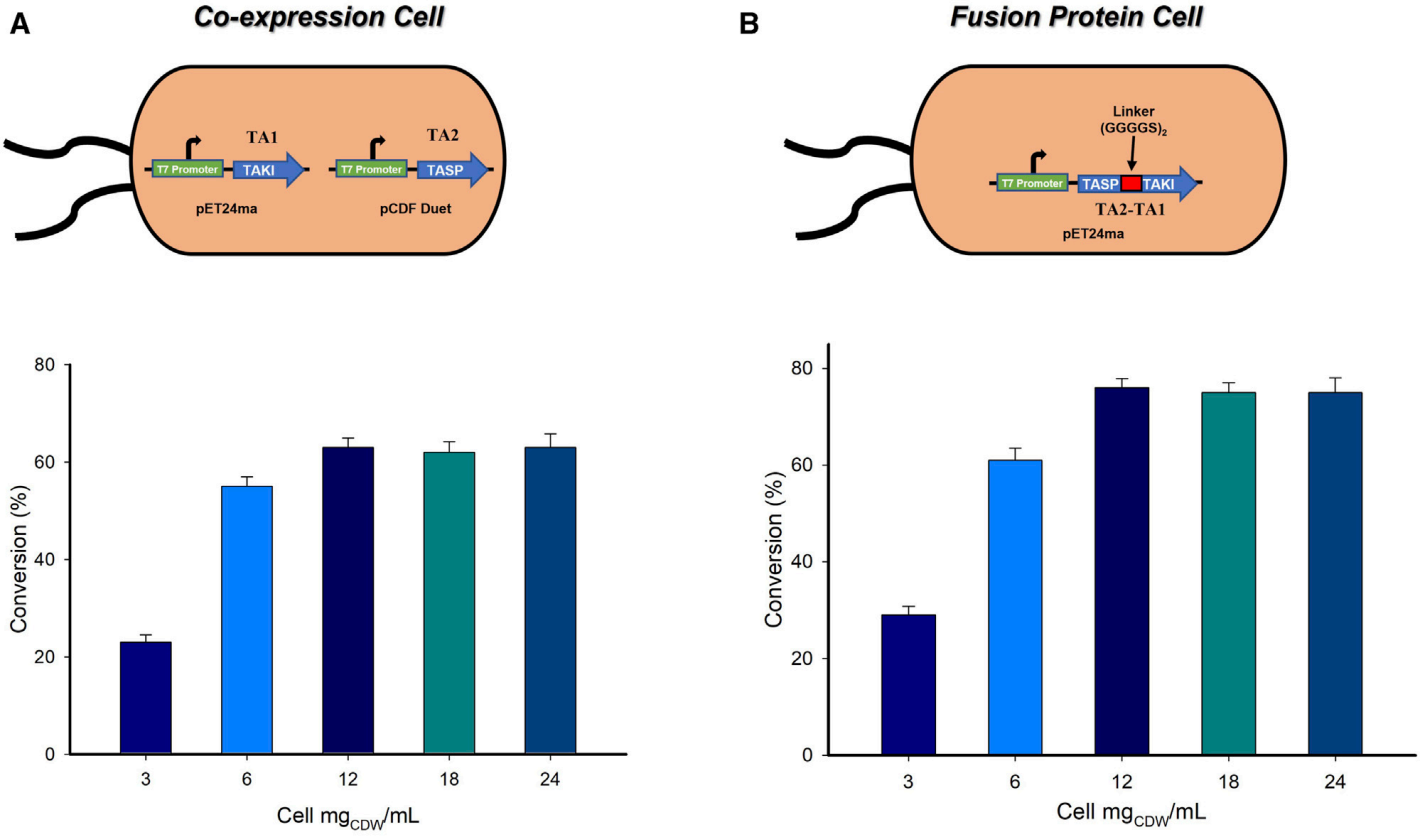
**(A) (A)** Effect of loading amount of whole-cells employing A3 system. Reaction conditions: 10 mM LA substrate, 0.1 mM PLP, 25 mM (*S*)-α-MBA, 100 mM IPA, 100 mM Tris-HCl buffer (pH 8.0), 37°C for 24 h. **(B)** Effect of loading amount of whole-cells employing A4 system. Reaction conditions: 10 mM LA substrate, 0.1 mM PLP, 25 mM (*S*)-α-MBA, 100 mM IPA, 100 mM Tris-HCl buffer (pH 8.0), 37°C for 24 h.

The ultimate outcomes of the biocatalytic cascade comprising more than a single whole-cell catalyst are often challenged by the mass transfer limitations (Patil et al., 2017; Patel et al., 2020). However, a single wholecell catalyst expressing multiple enzymes is easy to handle and less laborious, therefore reduced the overall cost of the enzymatic process and more importantly circumvent the mass-transfer limitations (Ahn et al., 2023). In order to address these issues, we developed a single-cell system co-expressing both the transaminases (System A3). Both TAs were initially cloned in pET-24ma vector. In order to generate a single-cell system, one of the TAs needed to be cloned in a different vector. Thus, TA_2_ was successfully cloned in the pCDF duet vector (Figure 4A). Having the single-cell expression system in hand, we further evaluated the effect of varying amounts of whole-cell catalyst on the formation of the desired product. A reaction was carried out using varying amounts of biocatalyst (3–24 mg_CDW_/mL), 10 mM substrate, 0.1 mM PLP, 25 mM (*S*)-α-MBA, 100 mM IPA, and 100 mM Tris-HCl buffer (pH 8.0) at 37°C for 24 h, wherein 12 mg_CDW_/mL of single-cell catalyst afforded the highest conversion of 62% (Figure 4A). On the other hand, the least conversion (21%) was observed with the use of 3 mg_CDW_/mL whole-cell catalyst.

In order to further improve the conversion of the LA substrate to the desired (*S*)-4-aminopentanoic acid, a fusion protein was constructed. Fusion proteins belong to a group of proteins that contain two or more distinct proteins combined into a single molecule, which normally demonstrate the enhanced enzyme expression, stability and catalytical activity. The selected both the TAs are dimeric enzymes. The original plasmid (pET24ma) encoding the TASP (TA_2_) on N-terminal and TAKI (TA_1_) on C-terminal served as the basis for recombinant proteins’ genetic assembly, and a flexible linker (GGGGS)_2_ was added to 2 TAs to aid in folding. It has been demonstrated that polar amino acids smaller in size, for example, glycine and serine offer the best flexibility and stability in water (Chen et al., 2013; Aalbers and Fraaje, 2019). After successful construction of a dual-function fusion protein (System A4) (Supplementary Figure S8), the effect of loading amount of whole-cell catalyst was evaluated by varying the amount of biocatalyst ranging from 3 to 24 mg_CDW_/mL in a reaction mixture containing 10 mM LA substrate, 0.1 mM PLP, 25 mM (*S*)-α-MBA, 100 mM IPA, 100 mM Tris-HCl buffer (pH 8.0), 37°C for 24 h (Figure 4B). The increase in the amount of whole-cells containing a fusion construct from 3 to 12 mg_CDW_/mL continuously increased the product formation and maximum conversion of 78% was achieved with the use of 12 mg_CDW_/mL whole-cells containing a fusion construct. This was a substantial increment of 20% compared to that achieved by the co-expressed single cell-system (System A3). Additionally, a time-point analysis was carried out using the 12 mg_CDW_/mL cells, which demonstrated the maximum conversion at 24 h (Supplementary Figure S9).

Furthermore, we investigated the impact of pH on the enzymatic process (Supplementary Figure S10). The experiments used 10 mM LA as the substrate and varied the pH between 7.0 and 10.0. While pH 7.0 showed the least conversion, pH 8.0 achieved the highest conversion of about 80%. On the other hand, product formation decreased as the pH rose to over 8.0. Thus, pH 8.0 was used for the next investigations. Furthermore, reactions with higher substrate concentrations (20 and 50 mM) were carried out, wherein mediocre conversions of only −15 and 0.5% were afforded (Supplementary Figure S11). These poor conversion rates could plausibly be attributed to the product and/or substrate inhibition. Thus, further efforts were dedicated to study the substrate and product inhibition.

To investigate the inhibition of fusion protein by LA substrate, a reaction was carried out for 30 min in 100 mM Tris−HCl buffer (pH 8.0) containing purified fusion protein (0.5 mg/mL), 25 mM (*S*)-α-MBA, 0.1 mM PLP and increasing concentration of substrate from 5 to 50 mM (Supplementary Figure S12A). Significant substrate inhibition was observed when the concentration of LA substrate exceeded 10 mM. Similar to this, a reaction was run for 30 min in a 100 mM Tris-HCl buffer (pH 8.0) containing purified fusion protein (0.5 mg/mL), 25 mM (*S*)-α-MBA, 0.1 mM PLP and in the presence of 5–50 mM (*S*)-4-aminopentanoic acid to evaluate the product inhibition of fusion protein by (*S*)-4-aminopentanoic acid (Supplementary Figure S12B). The results revealed that the purified fusion protein was substantially inhibited by (*S*)-4-aminopentanoic acid beyond the concentration of 10 mM.

### 3.4 Synthesis of dual function nanoflowers and their application for the enzymatic transamination reaction

**FIGURE 5:**
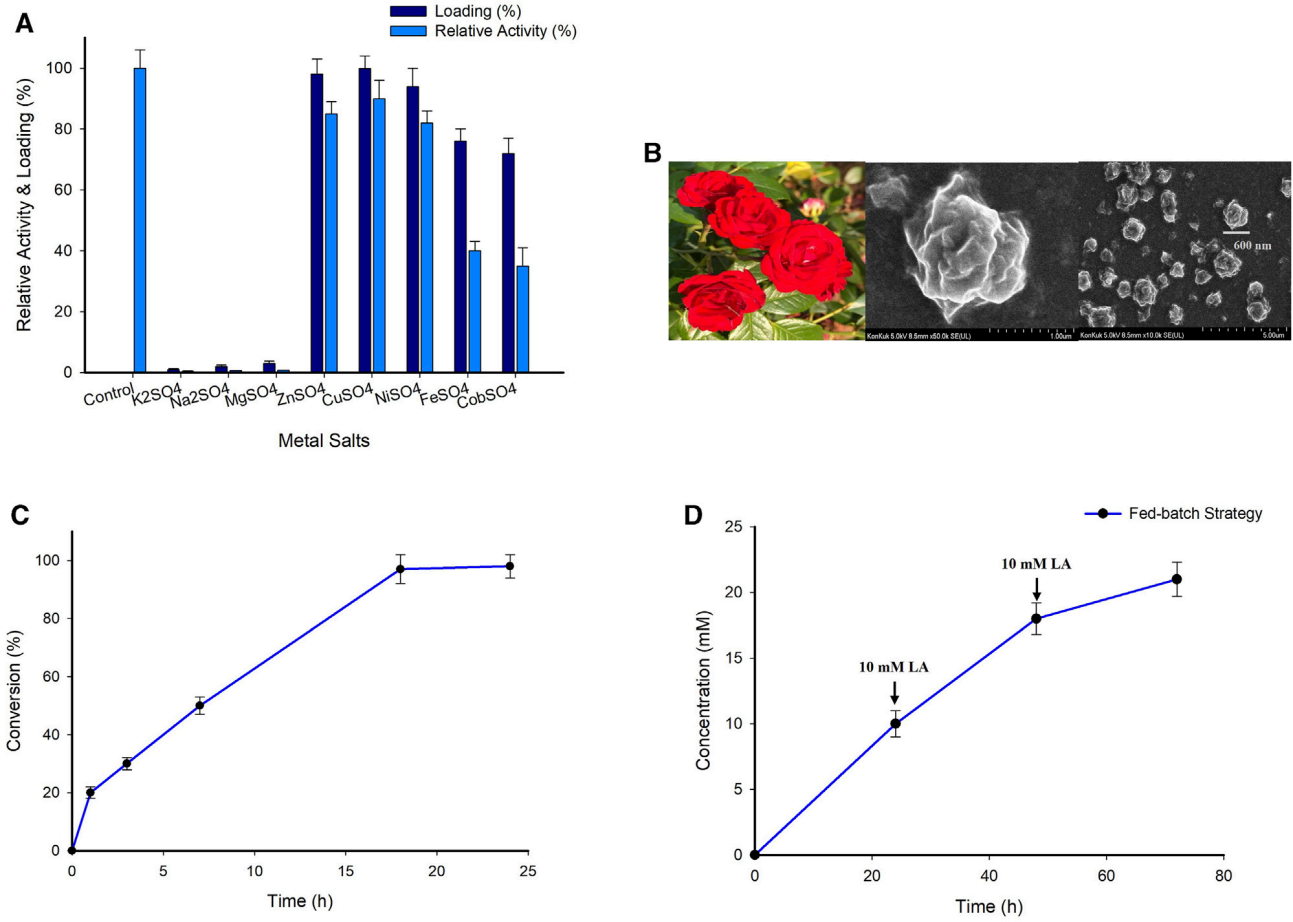
**(A)** Screening of various metal ions for the synthesis of nanoflowers. Reaction condition; 2 mM different metal salts, 0.5 mg/mL fusion protein 10 mM PBS buffer, 4°C, 72 h. **(B)** SEM image of hybrid nanoflowers. **(C)** Time course analysis for the synthesis (*S*)-4-aminopentanoic acid employing A7 system. Reaction condition; 10 mM substrate, 25 mM (*S*)-α-MBA, enzyme 0.5 mg/mL, 100 mM IPA, 0.1 mM PLP, 100 mM Tris-HCl buffer (pH 8.0), 37°C for 24 h. **(D)** Synthesis of (*S*)-4-aminopentanoic acid by employing fed-batch strategy. Reaction condition; 10 mM Substrate, 0.1 mM PLP, 25 mM (*S*)-α-MBA, 100 mM IPA, 0.5 mg/mL A7 system, 100 mM Tris-HCl buffer (pH 7.0–10.0), 37°C.

Hybrid nanoflowers (HNFs) are mixtures of organic and inorganic components with a three-dimensional hierarchical nanostructure resembling a flower (Li et al., 2020; Lv et al., 2020). Ge unintentionally mixed copper sulphate (CuSO_4_) into phosphate-buffered saline containing bovine serum albumin, leading to the discovery of the first HNF (Ge et al., 2012). HNFs have now garnered a lot of interest because of their ability to integrate the functions of organic and inorganic materials, as well as their quick and environmentally friendly preparation. In the present study, the self-assembly approach was used in the production of HNF, wherein the organic component was a fusion protein and the inorganic component was a metal salt. First, the efficiency of several salts including CuSO_4_, potassium sulphate (K_2_SO_4_), sodium sulphate (Na_2_SO_4_), magnesium sulphate (MgSO_4_), zinc sulphate (ZnSO_4_), nickel sulphate (NiSO_4_), ferrous sulphate (FeSO_4_) and cobalt sulphate (CoSO_4_) was investigated for the synthesis of HNFs. Nanoflowers were synthesized using CuSO_4_ salt displayed the maximum protein loading of about 99%, followed by 96% and 92% by ZnSO_4_ and NiSO_4_ salts, respectively. Whereas a moderate protein loading was observed for the FeSO_4_ and CoSO_4_. On the other hand, the K_2_SO_4_, Na_2_SO_4_, and MgSO_4_ salts, barely contained any protein loading. Subsequently, the effect of various metal ions on the enzyme activity was investigated. The relative activity was expressed as a percentage of the activity displayed by the fusion protein without immobilization (As a control experiment). While, the HNFs obtained using K_2_SO_4_, Na_2_SO_4_, and MgSO_4_ salts did not exhibit any activity, those obtained with CuSO_4_, ZnSO_4_, and NiSO_4_ salts maintained the relative activity around 80%–85%. In the case of FeSO_4_ and CoSO_4_ salts the relative activity of HNF exhibits 35%–38% (Figure 5A). Taking into account these findings, HNFs obtained with CuSO_4_, ZnSO_4_, and NiSO_4_ salts were selected for further research. The synthesized HNF has a rhombic shape having a dimension or size approx. 600 nm (Figure 5B). The Ge et al., 2012 reported the similar shape of HNF. However, we characterized the synthesized nanoflower using scanning electron microscopy (SEM).

Next, the HNFs obtained using CuSO_4_, ZnSO_4_, and NiSO_4_ salts were evaluated for the enzymatic conversion of the LA substrate. All the tested HNFs exhibited good conversions ranging from 82% to 100% (Supplementary Figure S13). While the HNF obtained using ZnSO_4_ and NiSO_4_ salts afforded conversions of 86% and 82% respectively, the complete conversion (>99%) was achieved by the HNF obtained using CuSO_4_ (Figure 5C). The conversion achieved by the CuSO_4_-HNF was 22% higher than that obtained by the fusion protein system (System A4). These positive results encouraged us to carry out the reactions with higher substrate concentrations (20 and 50 mM) (Supplementary Figure S14). Compared to the prior experiment, the product formation for the 20, 50 mM substrate reaction with HNF of CuSO_4_ increased by around 2.3 and 5-fold respectively. Employing HNF of ZnSO_4_ and NiSO_4_ salts also resulted in a modest conversion improvement. Additionally, the investigation of the enzymatic conversion of 10 mM LA substrate by employing purified protein TAKI and TASP (System A5) and purified fusion protein (System A6) resulted in the conversion of 82% and 85% respectively (Supplementary Figure S15). Owing to the better conversion afforded, HNF of CuSO_4_ salt (System A7) was used for further investigation.

In order to circumvent the limitations associated with product/substrate inhibition, we employed a fed-batch approach (Figure 5D). The enzymatic reaction employing A7 system was first carried out using 10 mM LA as a substrate. After the substrate had completely turned into a product in 24 h, 10 mM substrate was added again into the reaction which resulted in 8 mM product formation. Unfortunately, a further addition of 10 mM substrate resulted in conversion of only 3 mM. The overall conversion of about 70% was achieved by employing the fed-batch approach. As anticipated, the substrate and product inhibition experienced by the immobilized biocatalyst was slightly lessened compared to that by the free enzyme (Supplementary Figure S16).

**FIGURE 6:**
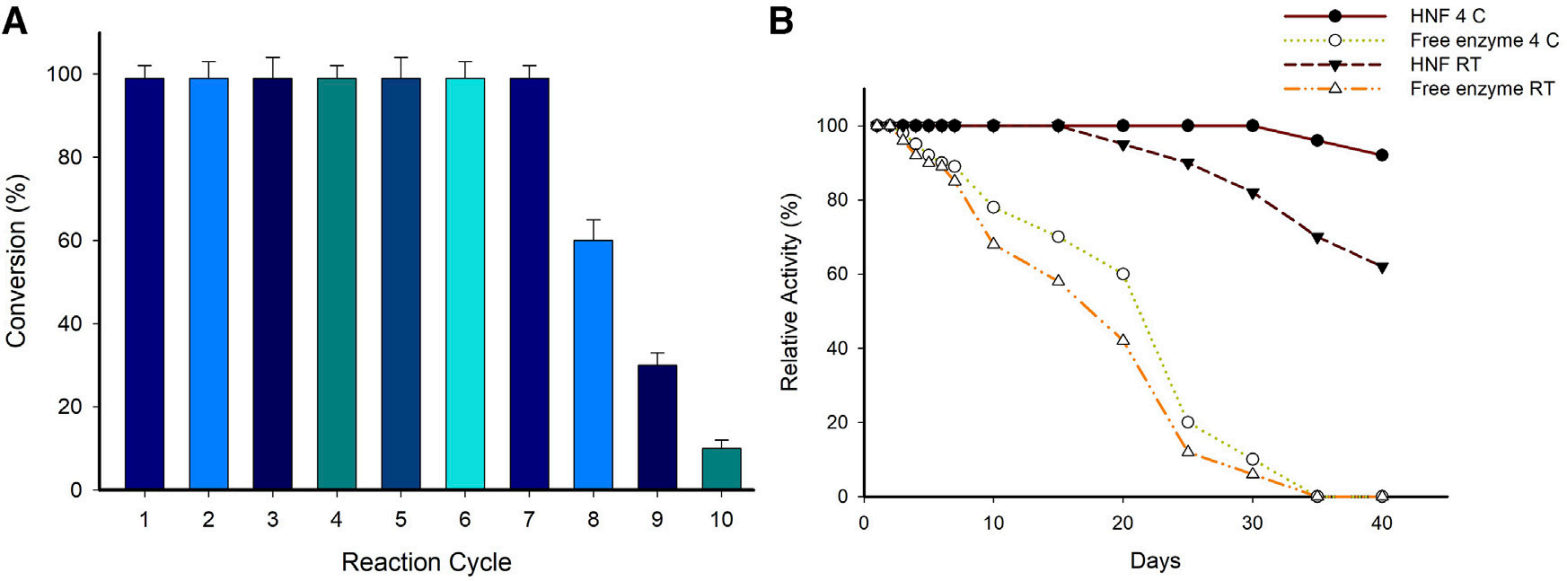
**(A)** Reusability of hybrid nanoflower (System A7) for the synthesis of (*S*)-4-aminopentanoic acid. Reaction conditions; 10 mM Substrate, 0.1 mM PLP, 25 mM (S)-α-MBA, 100 mM IPA, 3 mg/mL of system A7,100 mM Tris-HCl buffer (pH 8.0), 37°C, 24 h. **(B)** Storage stability of enzyme preparations at various temperatures. Reaction condition; 10 mM Substrate, 0.1 mM PLP, 25 mM (*S*)-α-MBA, 0.5 mg/mL A7, 100 mM Tris-HCl buffer (pH 8.0), 37°C for 30 min (all experiment performed in triplicate).

The operational stability is an important consideration in the economics of biotransformation’s involving immobilized biocatalysts. The repeated use of immobilized biocatalysts is one of the biggest advantages over the free enzyme. The hybrid nanoflowers synthesized using CuSO_4_ in the present study (System A7) could be utilized for seven consecutive cycles without any loss in activity (Figure 6A). The leaching of the enzyme from the hybrid nanoflowers and thermal deactivation during each batch reaction of 24 h can be attributed to the decreased conversion efficiency of hybrid nanoflowers after seven cycles. Evaluation of storage stability at different temperatures revealed that system A7 was stable for more than a month at 4°C and room temperature (Figure 6B). In contrast, the free enzyme lost more than 70% of its activity after 20 days when stored at different temperatures.

### 3.5 A preparative scale reaction, isolation of product and applicability of the dual-function hybrid nanoflowers for the synthesis of various β-amino acids

**FIGURE 7:**
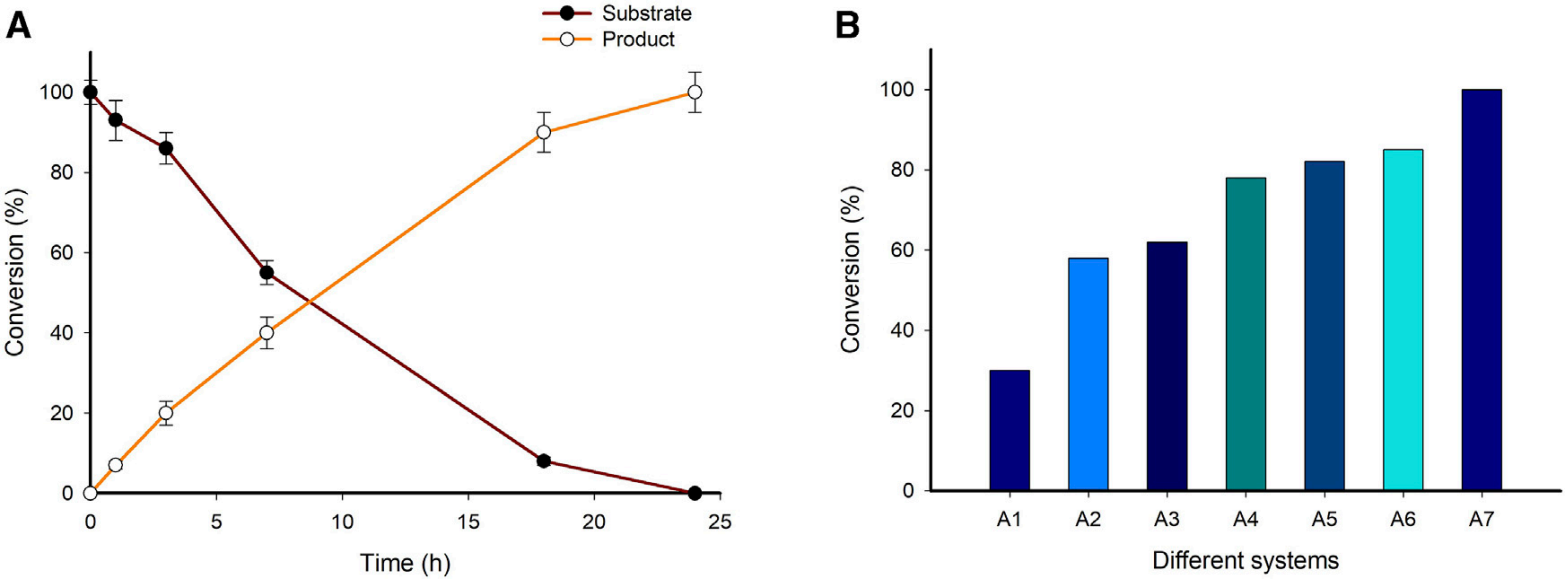
**(A)** Time course of preparative scale reaction for the synthesis of (*S*)-4-aminopentanoic acid from biobased LA. Reaction conditions; 10 mM Substrate, 0.1 mM PLP, 25 mM (*S*)-α-MBA, 100 mM IPA, 0.5 mg/mL HNF (System A7), 100 mM Tris-HCl buffer (pH 8.0), 37°C for 24 h (total volume 100 mL). **(B)** Elaborate figure to summarize the designed and developed systems for the synthesis of (*S*)-4-aminopentanoic acid from biobased LA. Reaction condition; 10 mM Substrate, 0.1 mM PLP, 25 mM (*S*)-α-MBA, 100 mM IPA, 100 mM Tris-HCl buffer (pH 8.0), 37°C for 24 h.

The synthetic applicability of the immobilized enzyme system (System A7) was investigated on a preparative scale (100 mg). The reaction mixture consisted of 10 mM LA substrate, 0.1 mM PLP, 25 mM (*S*)-α-MBA, 100 mM IPA, 0.5 mg/mL of System A7, 100 mM Tris-HCl buffer (pH 8.0), 37°C for 24 h in a total volume of 100 mL. The time-point analysis of samples showed almost complete consumption of substrate within 24 h, thereby achieving >99% conversion to the final product (*S*)-4-aminopentanoic acid (Figure 7A, Supplementary Figures S17, 18). After completion of the reaction, the reaction mixture was subjected to isolation and purification of the desired product (See material and method section). The isolated yield of desired product was 62% and characterized by using ^1^H NMR spectroscopy and HPLC (Supplementary Figures S19, 20). As we design and develop the different biocatalytic system for the production of (*S*)-4-aminopentanoic acid using biobased LA as a substrate the corresponding results summarized in Figure 7B.

β-amino acids are important structural moieties present in numerous natural and synthetic bioactive compounds (Jeon et al., 2018; Kim et al., 2019). The efficient synthesis of enantiopure (*S*)-4-aminopentoic acid by the biocatalytic system employing HNF encouraged us to check its applicability as a synthetic route for β-amino acids via asymmetric synthesis. Six β-keto ester substrates were tested for the synthesis of corresponding β-amino acids (Supplementary Figure S21 and Supplementary Table S4). Among the tested substrates, ethyl 3-(4-fluorophenyl)-3-oxopropanoate (1a) gave the highest conversion (48%). On the contrary, ethyl 3-(4-chlorophenyl)-3-oxopropanoate (1f) showed the least conversion (18%). The conversion of all the other β-keto ester substrates to their corresponding β-amino acids ranged from 30% to 40%. The conversion of aromatic β-amino acids was moderate with dual function HNF. However, TA_1_ was screened for LA as a substrate for the synthesis of (*S*)-4-aminopentoic acid not for the synthesis of aromatic β-amino acids. Therefore, it was not surprising to get less or poor conversion. Moreover, the dual function enzymes based HNF can enhance product formation compared to other developed systems. Furthermore, 1a was selected for the subsequent experiments (Supplementary Figure S22). Different cell systems previously developed for the synthesis of (*S*)-4-aminopentanoic acid were employed for the conversion of 1a to the corresponding β-amino acid (Supplementary Table S5). The whole cell biotransformation’s reaction was performed including a separately expressed 1 cells TA_1_ (System A1), separately expressed 2 cells TA_1_ and TA_2_ (System A2), 1 cells co-expressed 2 TAs (System A3) and 1 cells fusion proteins (system A4) and purified fusion protein system (System A5) were employed (Supplementary Table S5). While the separately expressed 1 cells TA_1_ (System A1) afforded only 8% conversion, the maximum conversion of 32%was achieved by the purified fusion protein system (System A5).

**FIGURE 8:**
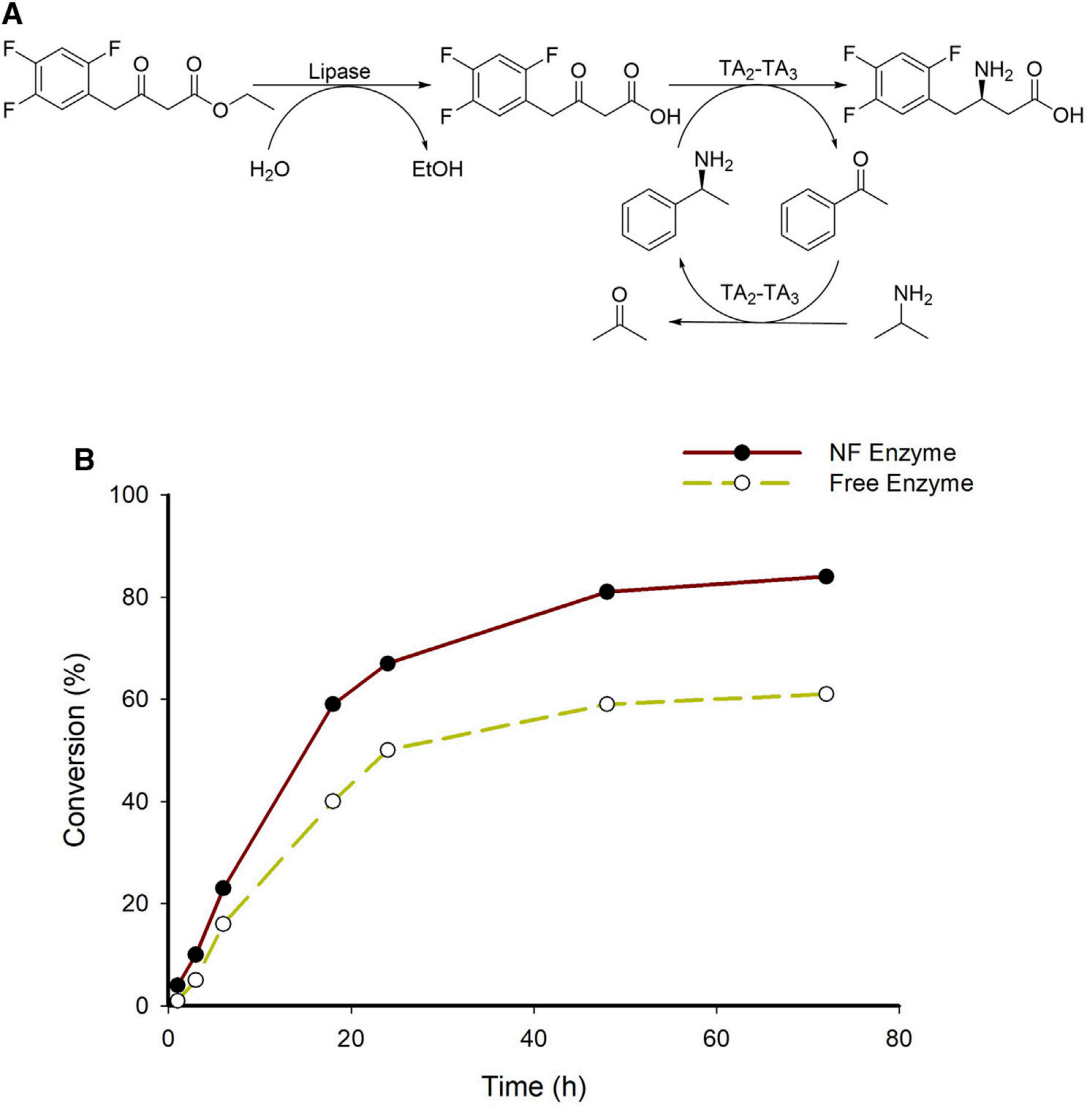
**(A)** A schematic representation of sitagliptin intermediate from corresponding β-keto ester substrate; **(B)** Time point analysis of sitagliptin intermediate from β-keto ester substrate employing A7 system. Reaction condition; 200 mM Substrate, 25 mM (*S*)-α-MBA, 15% DMSO, 0.5 mM PLP, 1 M IPA, 60 mg/mL lipase, 200 mM Tris-HCl buffer (pH 7.0), 37°C. #TA_3_-TAIC.

One of the well-known examples of medications with β-amino acids as essential ingredients is sitagliptin, an inhibitor of dipeptidyl peptidase 4 that is used as an oral diabetic medication (Savile et al., 2010; Kim et al., 2019; Khobragade et al., 2021; Kaur and Jana, 2023). In our previous research, the sitagliptin intermediate was produced employing TA from *Ilumatobacter coccineus* (TAIC, TA_3_) from the ethyl 3-oxo-4-(2,4,5-trifluorophenyl) butanoate as a substrate (Khobragade et al., 2023). Therefore, to test the applicability of developed system for the synthesis of sitagliptin intermediate here we employed TA_3_ (TAIC) instead of TA_1_ (TAKI) in a coupled transamination reaction (Figure 8A). A higher substrate enzymatic reaction (200 mM) was carried out, wherein a maximum conversion of 84% was achieved in 72 h (Figure 8B). Furthermore, we investigated a higher substrate concentration by employing a purified fusion protein system (System A6), which resulted in 67% of the final product. These findings indicated that the HNF system developed herein was a more efficient approach for the biosynthesis of various β-amino acids including sitagliptin intermediate.

## 4 Conclusion

In summary, we have successfully developed various biocatalytic systems to efficiently synthesize (*S*)-4-aminopentanoic acid from LA substrate. A recyclable cascade employing 2 TAs (TAKI and TASP) was developed for the co-product removal and recycling of the amino donor. Separately expressed one whole cells (TA_1_), separately expressed two whole cells (TA_1_ and TA_2_), one whole cells co-expressed system, and one whole cells fusion proteins were among the several whole cell systems established, wherein the purified fusion protein comprising of TAKI and TASP afforded 85% conversion of LA to (*S*)-4-aminopentanoic acid. The HNFs synthesized using this fusion protein construct improved the conversion by 3.3-fold compared to separately expressed 1 cells (TAKI) and retained the activity for 7 consecutive cycles while maintaining storage stability for more than a month. Preparative scale reaction (100 mL) using the HNFs achieved complete conversion with an isolated yield of 62%. Furthermore, the established biocatalytic system afforded the transformation of different β-keto ester substrates to corresponding β-amino acids with conversions ranging from 18% to 48%. Finally, the applicability of this established biocatalytic cascade was further extended for the enzymatic synthesis of sitagliptin intermediate with an excellent conversion of 82%. One of the features of this system is any TAs can utilize IPA as an amino donor through fused TA by a combination of another amino donor like (*S*)-α-MBA, L-alanine. The use of the catalytic amount of amino donors for recycling is crucial. Therefore, we devoted an effort to developing the system for the catalytic amount of amino donors under progress in our lab. In conclusion, we established the dual-function enzyme based one-pot cascade employing hybrid nanoflowers of fused two transaminase, which paves the way for expanding a toolbox for transaminases having difficulty to accept the isopropyl amine as an amino donor for the synthesis of various value-added amine compounds and would enjoy the extended applicability.

## Data Availability

The datasets presented in this study can be found in online repositories. The names of the repository/repositories and accession number(s) can be found in the article/Supplementary Material.
