# Supplementary material for: Dual-function transaminases with hybrid nanoflower for the production of value-added chemicals from biobased levulinic acid
Source: Front Bioeng Biotechnol. 2023 Nov 16;11:1280464. doi: 10.3389/fbioe.2023.1280464 (PMC10687574; doi:10.3389/fbioe.2023.1280464)
Supplement: Supplementary file 1 [file DataSheet1.docx]

Supplementary Material

# Supplementary Data

**Material and Method**

**Whole cell reaction employing different cell system.**

The biocatalytic reactions containing a 10 mM LA as substrate, 100 mM IPA, 25 mM (*S*)-α-MBA, 0.1 mM PLP, 100 mM Tris-HCl buffer (pH 8.0) and separately expressed, co-expressed or fusion cell TAs in a total volume of 500 μL. The reactions were performed for 24 h and samples were taken at specific time intervals and analyzed using HPLC.

**Activity assay and enzymatic synthesis using hybrid nanoflowers.**

To determine the specific activity of the enzyme, a reaction was carried out in a total volume of 500 µL containing Tris-HCl buffer (100 mM, pH 8.0), LA (10 mM) as a substrate, (*S*)-α-MBA (25 mM), PLP (0.1 mM), enzyme (0.5 mg/mL) at 37 °C for 30 min with 180 rpm. The reaction was stopped by adding 10% perchloric acid in a ratio of 1:1 (v/v). After centrifuging the reaction solution at 17000 g for 30 min, subsequently the clear supernatant was subjected to HPLC analysis. One unit of enzyme activity was defined as the amount of enzyme required to generate 1 μmol of the corresponding product per minute.

**Analytical methods**

HPLC was used to measure the conversion of LA at 210 nm with an Aminex column and 5 mM H2SO_4_ as the mobile phase at a flow rate of 0.7 mL/min. As previously reported, a Crownpak CR column from Daicel Co. in Japan was used for the quantitative measurement of β and γ-amino acids. Perchloric acid solution (pH 1.5 or 1.0; 0.6 or 0.3 mL/min) was used as the elution solvent. Amine compound was analyzed by slight modification in this method a mixture of 300 μl borate buffer (50 mM, pH 9), 100 μl methanol, 47 μl distilled water, 50 μl target sample, and 3 μl DEEMM. The derivatization reaction was performed using 500 μl at 70°C for 2 h to derivatize amine. Analysis was performed after derivatization using C_18_ column at 284 nm. The mobile phase was composed of 100% acetonitrile (solvent A) and 25 mM sodium acetate buffer pH 4.8 (solvent B). The flow rate was maintained at 1 ml/min and the composition of solvent A to B (A:B, v/v) was changed with the following gradient program: 0 min (20:80), 2 min (25:75), 20 min (28:72), 25 min (20:80).

# Supplementary Figures and Tables

## Supplementary Figures


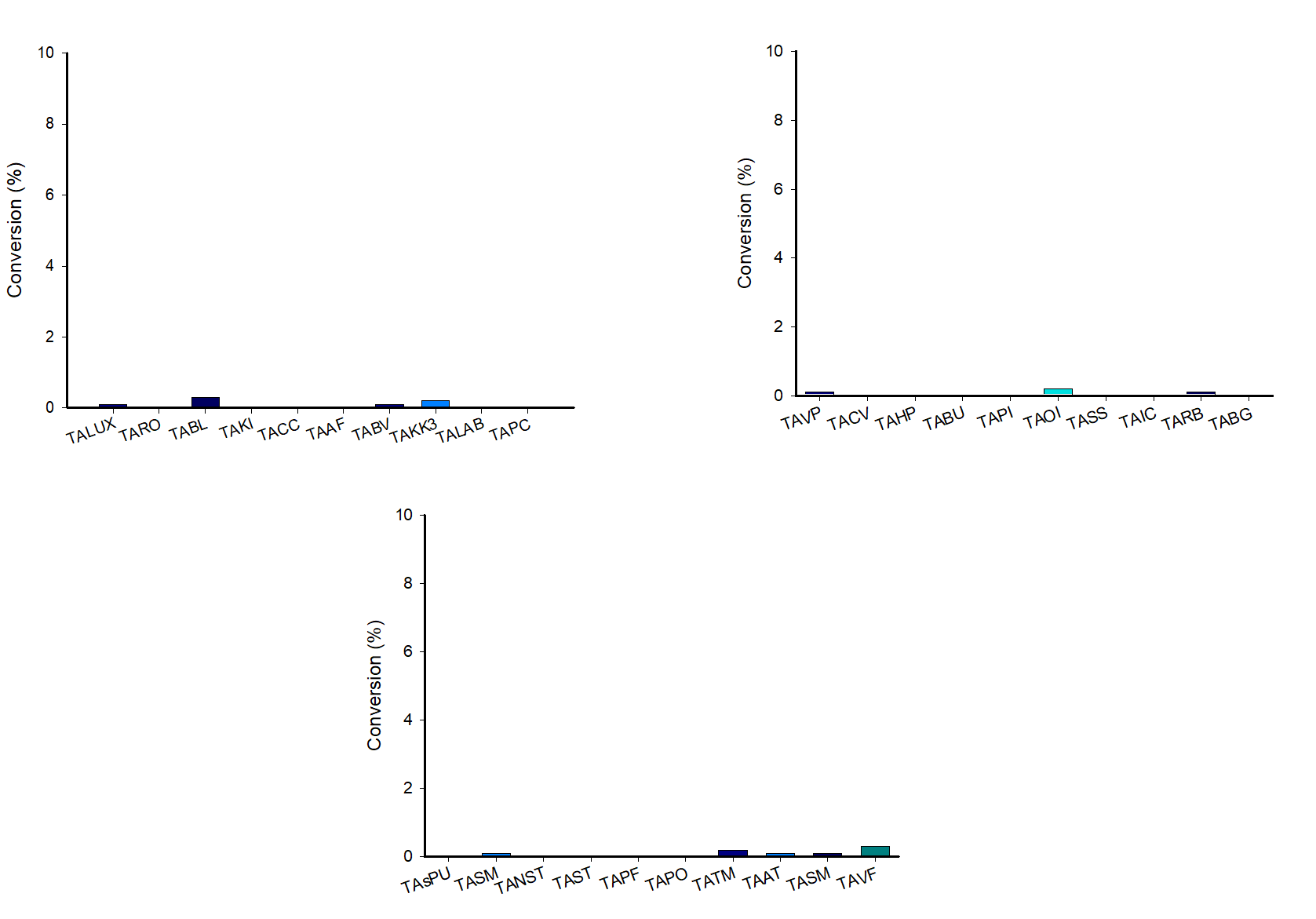


**Supplementary Figure 1.** Screening of TAs for the amination of LA using IPA as an amino donor. Reaction conditions: 10 mM LA as a substrate, 100 mM IPA, 0.1 mM PLP, and 100 mM Tris-HCl buffer pH 8.0 at 37 °C for 24 h.


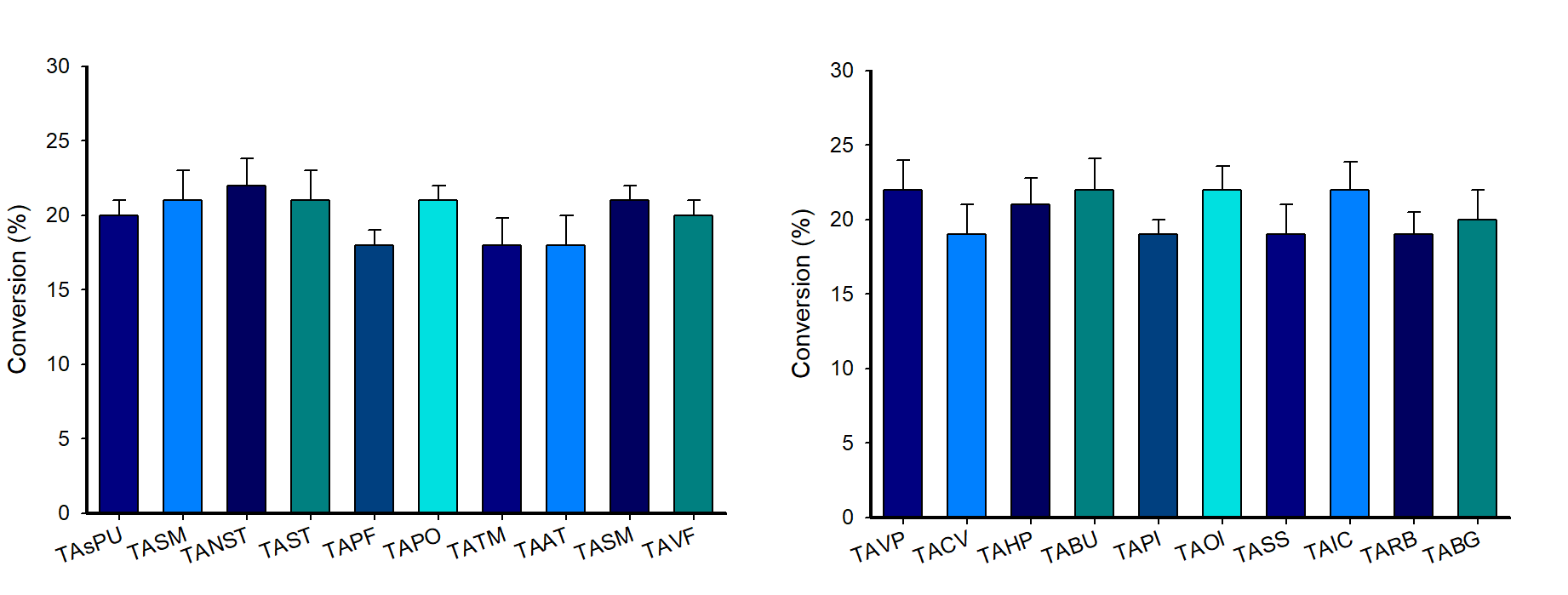


**Supplementary Figure 2.** Screening of TAs for the amination of LA using (*S*)-α-MBA as an amino donor. Reaction conditions: 10 mM LA as a substrate, 25 mM (*S*)-α-MBA, 0.1 mM PLP, and 100 mM Tris-HCl buffer pH 8.0 at 37 °C for 24 h.


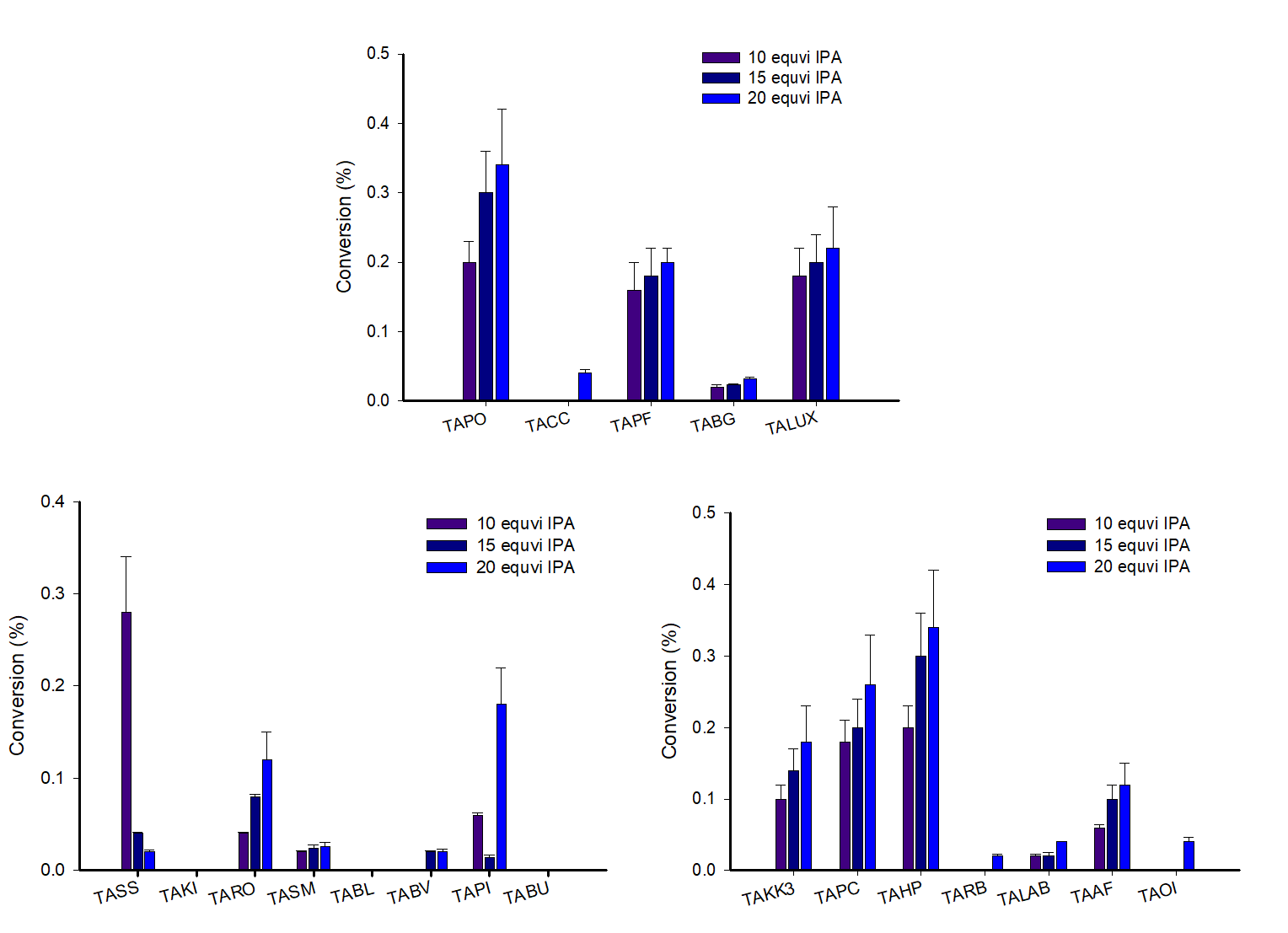


**Supplementary Figure 3.** Screening of TAs for the conversion of acetophenone to (*S*)-MBA using isopropyl amine as an amino donor. Reaction condition; 50 mM acetophenone substrate, IPA (10, 15, or 20 equivalent) 0.5 mM PLP, 12 mg_CDW_/ml cells of each TA, 37°C for 24 h.

**Supplementary Figure 4.** Comparison of TAs and alanine dehydrogenase amino donor recycling system. Reaction conditions: 10 mM LA as a substrate, 25 mM (*S*)-α-MBA or L-alanine, 100 mM IPA, 0.1 mM PLP, 6 mg_CDW_/mL of cell TA_1_ and 6 mg_CDW_/mL of cell TA_2_ or 6 mg_CDW_/mL of cell AlaDH and 6 mg_CDW_/mL of cell FDH, 100 mM sodium formate and 100 mM Tris-HCl buffer pH 8.0 at 37 °C for 24 h.

**Supplementary Figure 5.** Comparison of MBA and IPA as an amino donor using TA_1_ transaminase. Reaction conditions: 10 mM LA as a substrate, 25 mM (*S*)-α-MBA or 100 mM IPA, 0.1 mM PLP, 6 mg_CDW_/mL of cell TA_1_ and 100 mM Tris-HCl buffer pH 8.0 at 37 °C for 24 h.

**Supplementary Figure 6.** Comparison of reported and newly screened transaminase. Reaction Condition: 10 mM substrate, 25 mM (*S*)-α-MBA, 0.1 mM PLP, 100 mM Tris-HCl buffer (pH 8.0), 37 °C for 24 h.

**Supplementary Figure 7.** Optimization of amino donor employing separately expressed one transaminase (TAKI). Reaction Condition: 10 mM substrate, 25 mM (*S*)-α-MBA, 0.1 mM PLP, 100 mM Tris-HCl buffer (pH 8.0), 37 °C for 24 h.


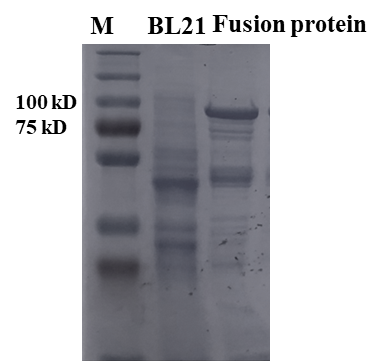


**Supplementary Figure 8.** SDS PAGE Gel of fusion protein (M-marker, BL21 cell, Fusion protein).

**Supplementary Figure 9.** Time point analysis for the synthesis of (*S*)-4-aminopentanoic acid employing System A4. Reaction conditions: 10 mM LA as a substrate, 25 mM (*S*)-α-MBA, 100 mM IPA, 0.1 mM PLP, 12 mg_CDW_/mL of cell System A4 and 100 mM Tris-HCl buffer pH 8.0 at 37 °C for 24 h.

**Supplementary Figure 10.** The synthesis of (*S*)-4-aminopentanoic acid using A4 system at different pH. Reaction conditions: 10 mM substrate, 25 mM (*S*)-α-MBA, 100 mM IPA, 0.1 mM PLP, 12 mg_CDW_/mL of cell, 37 °C for 24 h.

**Supplementary Figure 11.** Synthesis of (*S*)-4-aminopentanoic acid using higher substrate concentrations by employing A4 system. Reaction conditions: 0.1 mM PLP, 25 mM (*S*)-α-MBA, 200 or 500 mM IPA, 24 mg_CDW_/mL cell of A4 system, 100 mM Tris-HCl buffer (pH 8.0), 37 °C for 24 h.


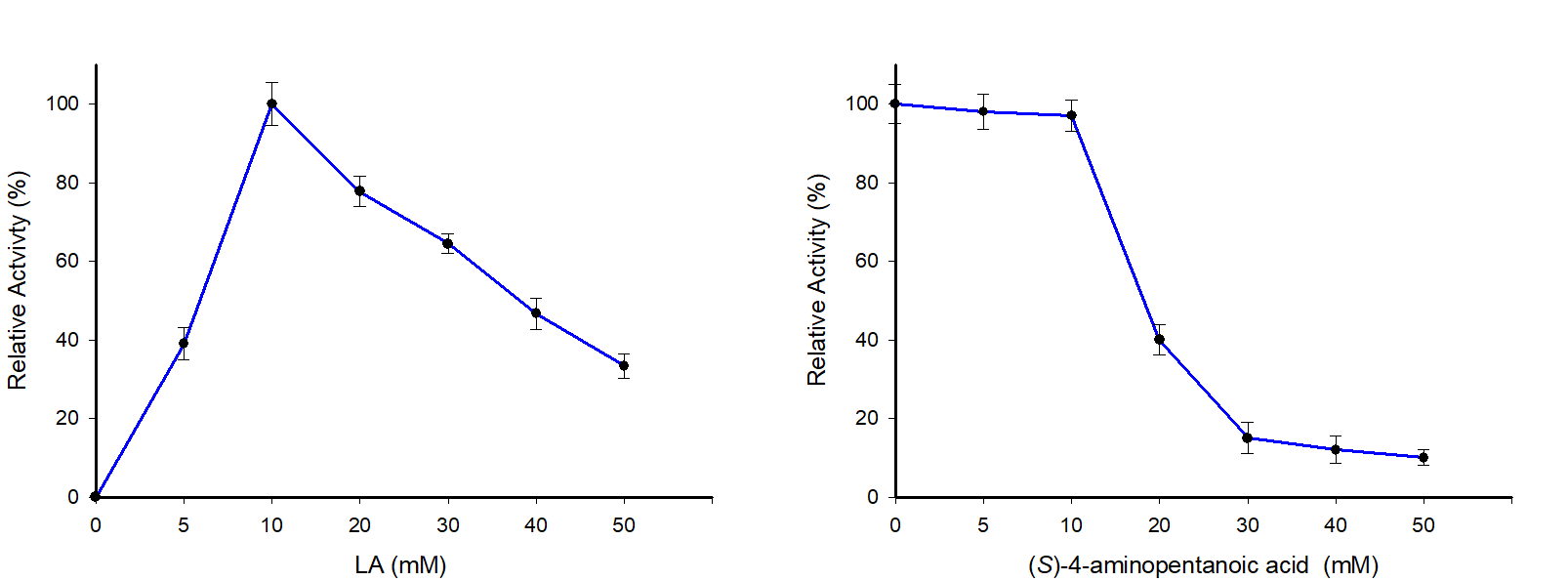


**Supplementary Figure 12.** A) Substrate inhibition assay. Reaction conditions: 5-50 mM LA substrate, 0.1 mM PLP, 25 mM (*S*)-α-MBA, 0.5 mg/mL fusion protein,100 mM Tris-HCl buffer (pH 8.0), 37 °C for 30 min. B) Product inhibition assay. Reaction condition; 5-50 mM (*S*)-4-aminopentanoic acid, 0.1 mM PLP, 25 mM (*S*)-α-MBA, 0.5 mg/mL fusion protein ,100 mM Tris-HCl buffer (pH 8.0), 37 °C for 30 min.

**Supplementary Figure 13.** Enzymatic synthesis of (*S*)-4-aminopentanoic acid using nanoflower of CuSO_4_, ZnSO_4_ and NiSO_4_ (fusion protein). Reaction conditions: 10 mM LA, 0.1 mM PLP, 25 mM (*S*)-α-MBA, 100 mM IPA, 0.5 mg/mL of fusion protein (CuSO_4_, ZnSO_4_ and NiSO_4_), 100 mM Tris-HCl buffer (pH 8.0), 37 °C for 24 h.

**Supplementary Figure 14.** Synthesis of (*S*)-4-aminopentanoic acid using higher substrate concentrations by employing nanoflowers obtained using CuSO_4_. Reaction conditions: 0.1 mM PLP, 25 mM (*S*)-α-MBA, 200 or 500 mM IPA, 0.5 mg/mL of A7, 100 mM Tris-HCl buffer (pH 8.0), 37 °C for 24 h.

**Supplementary Figure 15** Enzymatic synthesis of (*S*)-4-aminopentanoic acid employing purified protein TAKI and TASP (System A5) and purified fusion protein (System A6). Reaction condition; 10 mM LA, 0.1 mM PLP, 25 mM (*S*)-α-MBA, 100 mM IPA, 0.5 mg/mL of purified fusion protein, 100 mM Tris-HCl buffer (pH 8.0), 37 °C for 24 h.


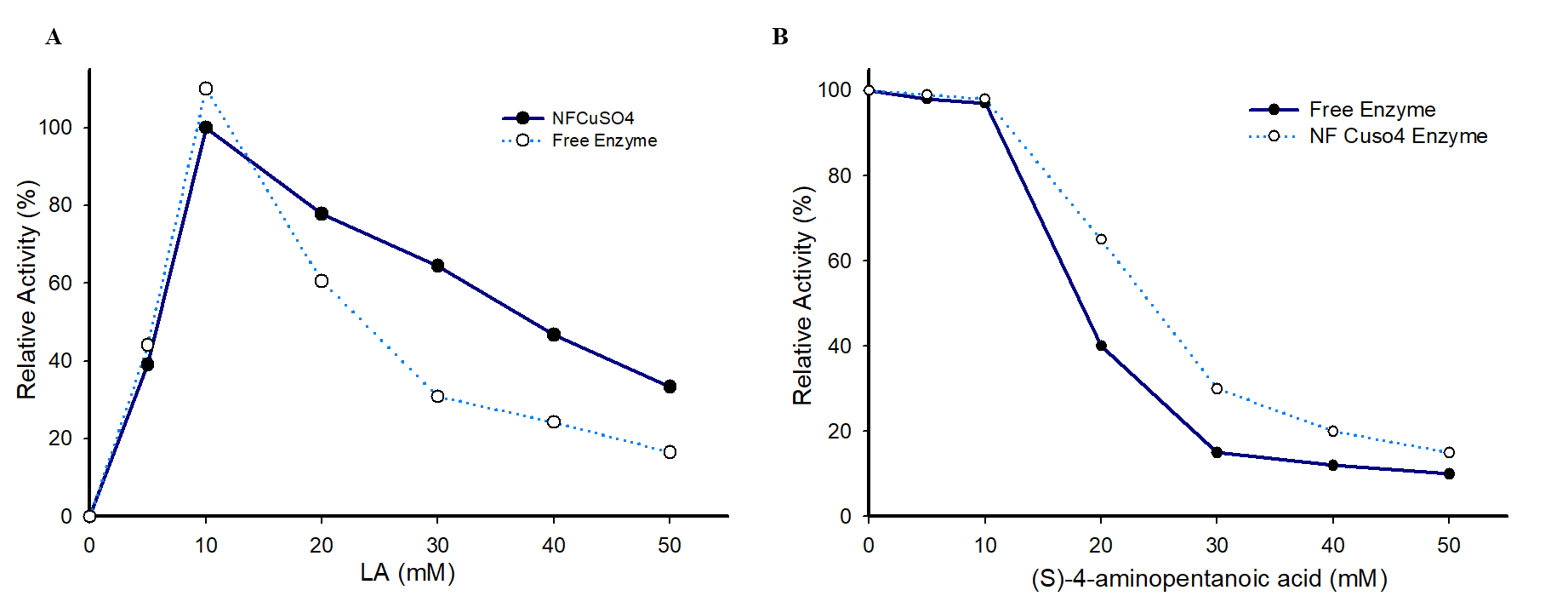


**Supplementary Figure 16.** Substrate and product inhibition assays employing purified enzyme and A7 system. A) Substrate inhibition assay. Reaction conditions: 5-50 mM LA substrate, 0.1 mM PLP, 25 mM (S)-α-MBA, 0.5 mg/mL fusion protein,100 mM Tris-HCl buffer (pH 8.0), 37 °C for 30 min. B) Product inhibition assay. Reaction condition; 5-50 mM (S)-4-aminopentanoic acid, 0.1 mM PLP, 25 mM (S)-α-MBA, 0.5 mg/mL fusion protein ,100 mM Tris-HCl buffer (pH 8.0), 37 °C for 30 min. (All experiment was performed in triplicate)


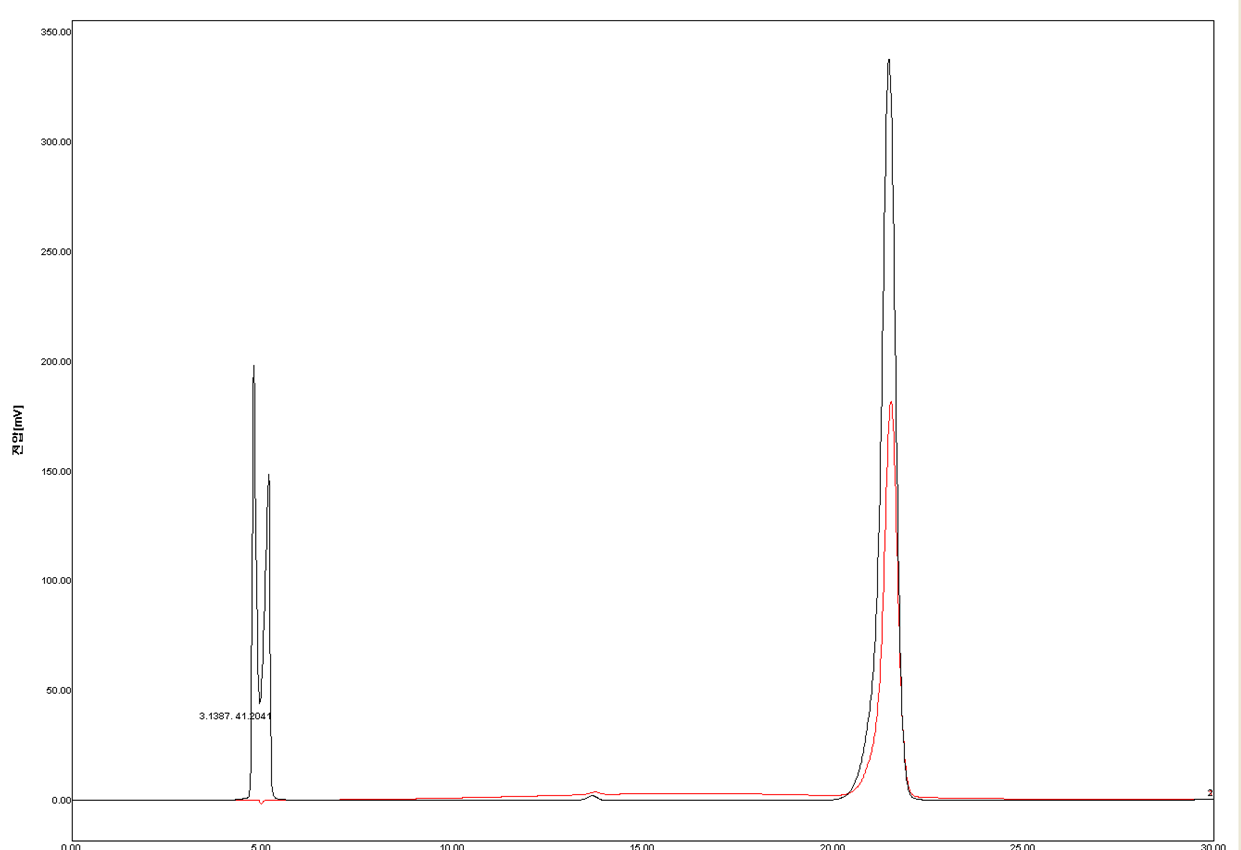


**Supplementary Figure 17.** HPLC chromatogram of LA (red); reaction sample (black).


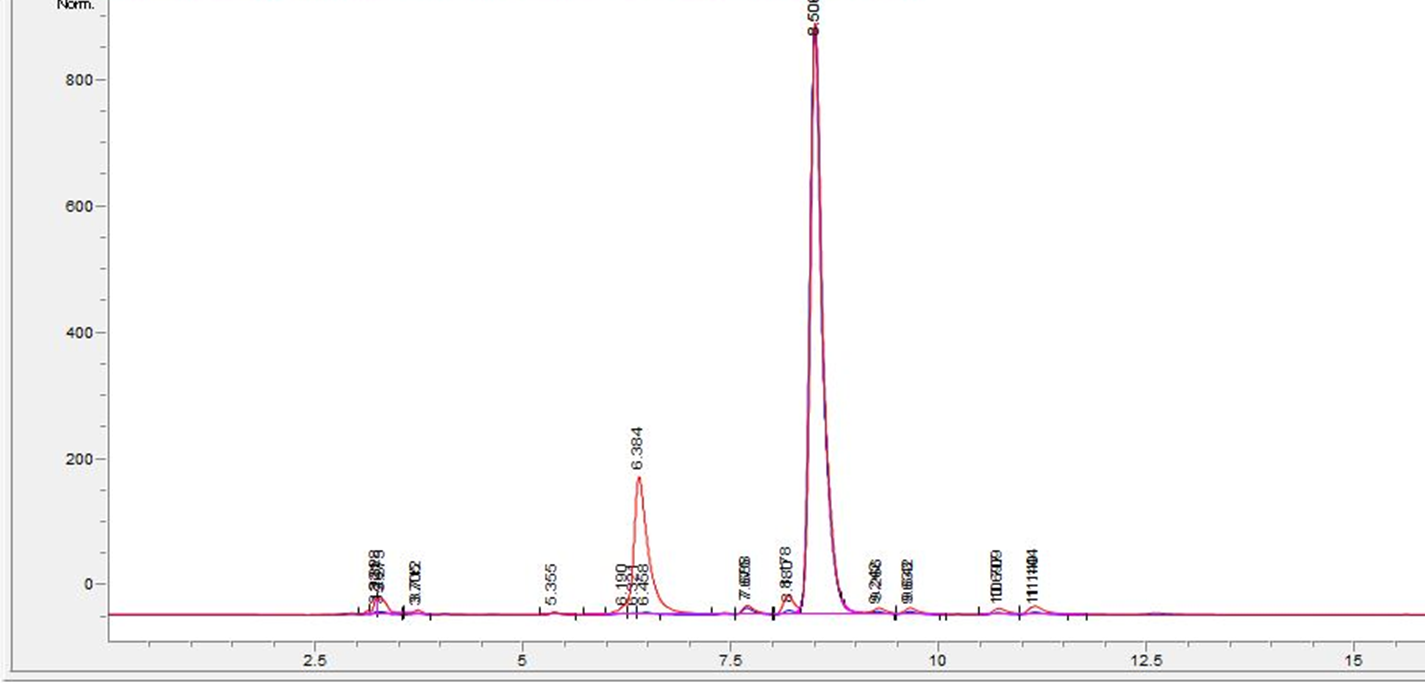


**Supplementary Figure 18.** HPLC chromatogram of (*S*)-4-aminopentanoic acid (pink); reaction sample (red).


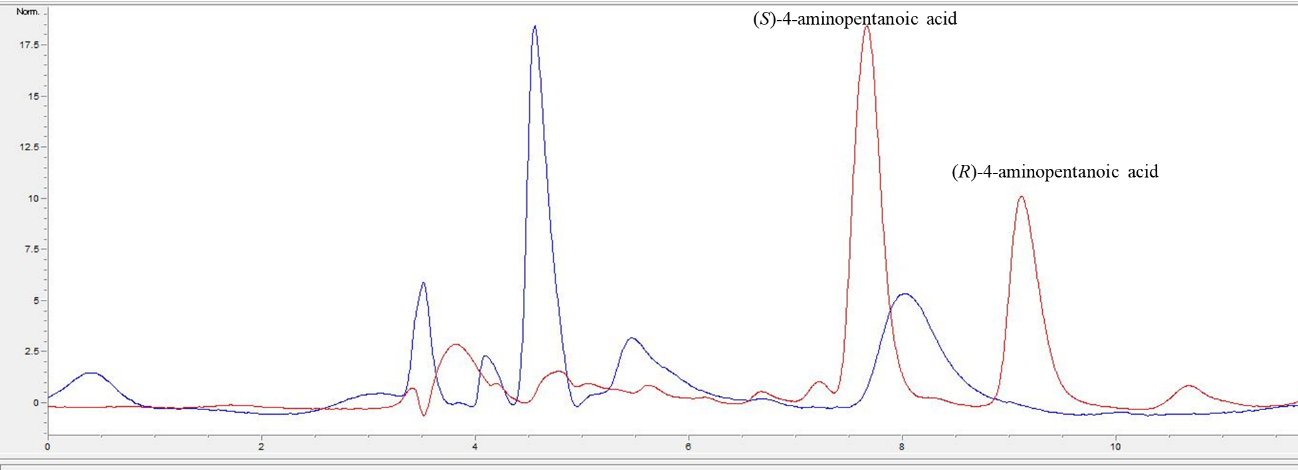


**Supplementary Figure 19.** HPLC chromatogram of 4-aminopentanoic acid (red); reaction sample (blue).


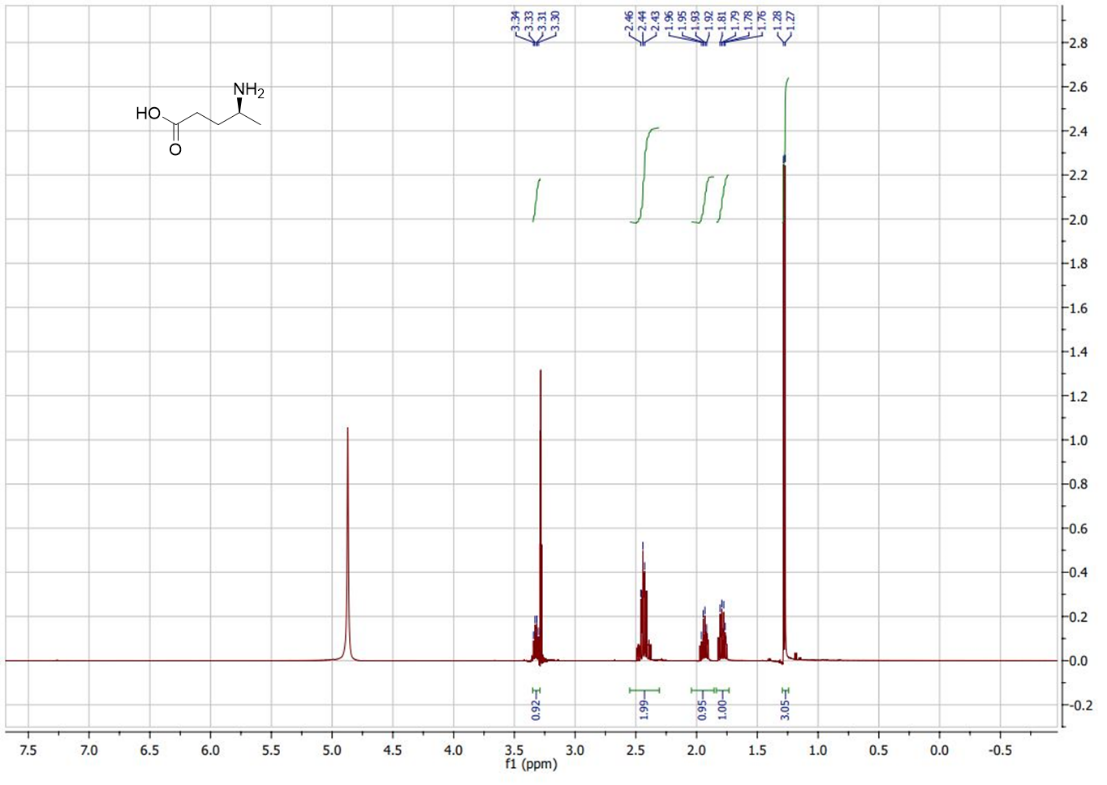


**Supplementary Figure 20.** White solid; ^1^H NMR (400 MHz, Methanol-d4): 3.32 (dd, *J* = 4 Hz, *J* = 12 Hz, 1H), 2.46-2.43 (m, 2H), 1.94 (dd, *J* = 4 Hz, *J* = 12 Hz, 1H), 1.28 (d, *J* = 4 Hz, 3H).

**Supplementary Figure 21.** Reaction scheme for the synthesis of different β-amino acids via asymmetric synthesis.


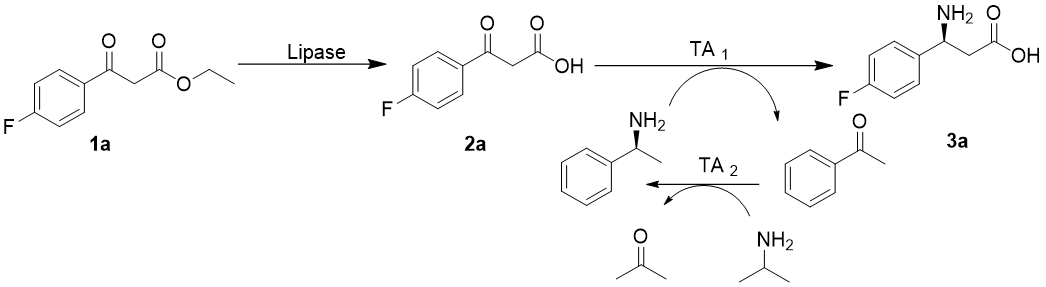


**Supplementary Figure 22.** Reaction scheme for the synthesis β-amino acids via asymmetric synthesis.

## Supplementary Tables

**Supplementary Table 1**. Enzymes used in this study.

| Entry | Enzyme | Assigned name | GenBank identification number | Reference |
| --- | --- | --- | --- | --- |
| 1 | *Rhodospirillaceae bacterium* | TARB | WP 092830126.1 |  |
| 2 | *Labrenziasp-*LAB | TALAB | WP 0841772225.1 |  |
| 3 | *Afipia* sp. P52-10 | TAAF | WP 051444297.1 |  |
| 4 | *Oceanibaculum indicum* | TAOI | WP 008945048.1 |  |
| 5 | *Ilumatobacter coccineus* | TAIC | WP 015443725.1 |  |
| 6 | *Variovorax* sp. KK3 | TAKK_3_ | WP 076998155.1 |  |
| 7 | *Paraburkholderia caribensis* | TAPC | AMV 45988.1 |  |
| 8 | *Hydrogenophaga palleronii* | TAHP | WP 066271212.1 |  |
| 9 | *Solirubrobacter soli* | TASS | WP 028067622.1 |  |
| 10 | *Kineosporia* sp. *R_H_3* | TAKI | WP 088319003.1 |  |
| 11 | *Roseomonas deserti* | TARO | WP 076960246.1 |  |
| 12 | *Sinorhizobium meliloti* | TASM | WP 018099655.1 |  |
| 13 | *Bosea lupine* | TABL | WP 091836827.1 |  |
| 14 | *Bosea vaviloviae* | TABV | WP 069688334.1 |  |
| 15 | *Pseudacidovorax intermedius* | TAPI | WP 052383549.1 |  |
| 16 | *Burkholderia* sp. *UYPR1.413* | TABU | WP 028368728.1 |  |
| 17 | *Synechocystis* sp. | AHR | WP_028949165.1 |  |
| 18 | *Pseudomonas* sp. *101* | FDH | P33160.3 |  |
| 19 | *Bacillus* | AlaDH | WP003243280.1 | Kim et al., 2019 |
| 20 | *Variovorax Paradoxus* | TAVP | WP_013541041.1 |  |
| 21 | *Coccidioides posadasii C735 delta SOWgp* | TACC | XP_003065334.1 |  |
| 22 | *Pseudomonas fluorescens WH6* | TAPF | EFQ61134.1 |  |
| 23 | *Sphaerobacter Thermophilus* | TAST | WP_012871332.1 |  |
| 24 | *Polaromonas sp. JS666* | TAPO | WP_011482414.1 |  |
| 25 | *Vibrio Fluvialis* | TAVF | 3NUI_A |  |
| 26 | *Thermomicrobium Roseum* | TATM | WP_015922462.1 |  |
| 27 | *Agrobacterium Tumefaciens* | TAAT | WP_060724765.1 |  |
| 28 | *Mesorhizobium Loti* | TAML | WP_010909990.1 |  |
| 29 | *Sphaerobacter thermophilus* | TAHST | WP_012872904.1 |  |
| 30 | *Mesorhizobium sp. LUK* | TALUX | WP_245488518.1 |  |
| 31 | *Pseudomonas putida* | TASPU | WP_016502144.1 | Mathew et al., 2016 |
| 32 | *Chromobacterium violaceum* | TACV | WP_011135573.1 | Kaulmann et al., 2007 |
| 33 | *Silicibacter pomeroyi* | TASP | WP_011049154.1 | Sung et al., 2018 |
| 34 | *Burkholderia vietnamiensis* | TABKV | YP_001110355.1 | Jiang et al., 2014 |

**Supplementary Table 2.** Enzymatic reaction using separately expressed one and two transaminases.

| **Enzymes** | **LA Substrate (mM)** | **(*S*)-α-MBA (mM)** | **IPA (mM)** | **Product formed (%)** |
| --- | --- | --- | --- | --- |
| TA_1_ (TARO) | 10 | 25 | - | 23 |
| TA_1_/TA_2_ (TARO/TASP) | 10 | 25 | 100 | 39 |
| TA_1_ (TAKI) | 10 | 25 | - | 31 |
| TA_1_/TA_2_ (TAKI/TASP) | 10 | 25 | 100 | 58 |
| TA_1_ (TAPI) | 10 | 25 | - | 22 |
| TA_1_/TA_2_ (TAPI/TASP) | 10 | 25 | 100 | 32 |
| TA_1_ (TAAF) | 10 | 25 | - | 29 |
| TA_1_/TA_2_ (TAAF/TASP) | 10 | 25 | 100 | 42 |
| TA_1_ (TABV) | 10 | 25 | - | 26 |
| TA_1_/TA_2_ (TABV/TASP) | 10 | 25 | 100 | 40 |

Reaction condition; 10 mM LA, 25 mM (*S*)-α-MBA, 100 mM IPA, 0.1 mM PLP, 6 mg_CDW_/mL of cell TA_1_ and 6 mg_CDW_/mL of cell TA_2_, 100 mM Tris-HCl buffer (pH 8.0), 37 °C for 24 h.

**Supplementary Table 3**. Kinetic parameter of new screened TA and reported TA.

| **Enzymes** | **K_M_ (mM)** | **k_cat_ (sec^-1^)** | **k_cat_/K_M_ (mM^-1^ sec^-1^)** |
| --- | --- | --- | --- |
| TAKI | 5.9 | 2.46 | 0.41 |
| TABKV | 6.8 | 0.84 | 0.12 |

Reaction Condition: 0-30 mM substrate, 25 mM (S)-α-MBA 0.1 mM PLP, 100 mM Tris-HCl buffer (pH 8.0), 37 °C for 30 min. (All experiment was performed in triplicate)

**Supplementary Table 4**. Synthesis of different β-amino acids via asymmetric synthesis.

| **Entry** | **Substrate** | **Conversion (%)** |
| --- | --- | --- |
| 1 | 1a | 48 |
| 2 | 1b | 41 |
| 3 | 1c | 30 |
| 4 | 1d | 28 |
| 5 | 1e | 27 |
| 6 | 1f | 18 |

Reaction Condition: 10 mM Substrate (See Supplementary Figure 20), 0.1 mM PLP, 25 mM (*S*)-α-MBA, 100 mM IPA, 0.5 mg/mL of A7 system, 10 mg/mL lipase, 100 mM Tris-HCl buffer (pH 8.0), 37 °C for 24 h.

**Supplementary Table 5**. Synthesis of β-amino acid (3a) using a different developed system.

| **Developed System** | **Substrate (1a) (mM)** | **(*S*)-α-MBA (mM)** | **IPA (mM)** | **Conversion (%)**  **(3a)** |
| --- | --- | --- | --- | --- |
| A1 | 10 | 25 | - | 8 |
| A2 | 10 | 25 | 100 | 18 |
| A3 | 10 | 25 | 100 | 20 |
| A4 | 10 | 25 | 100 | 24 |
| A5 | 10 | 25 | 100 | 32 |
| A7 | 10 | 25 | 100 | 48 |

Reaction Condition: 10 mM Substrate (See Supplementary Figure 21), 0.1 mM PLP, 10 mg/mL lipase, 100 mM Tris-HCl buffer (pH 8.0), 37 °C for 24 h.

**References**

Khobragade, T.P., Yu, S., Jung, H., Patil, M.D., Sarak, S., Pagar, A.D., Jeon, H., Lee, S., Giri, P., Kim, G-H., Cho, S.S., Park, S.H., Park, H.J., Kang, H.M., Lee, S.R., Lee, M.S., Kim, J.H., Choi, I.S., Yun, H. (2021). Promoter Engineering‐ mediated Tuning of Esterase and Transaminase Expression for the Chemoenzymatic Synthesis of Sitagliptin Phosphate at the kilogram‐scale. *Biotechnol. Bioeng*, 118(8), 3263-3268. doi.org/10.1002/bit.27819

Kim, G.H., Jeon, H., Khobragade, T.P., Patil, M.D., Sung, S., Yoon, S., Won, Y., Choi, I.S., Yun, H. (2019). Enzymatic synthesis of sitagliptin intermediate using a novel ω‐transaminase. *Enzyme Microb. Technol*, 120, 52–60. doi.org/10.1016/j.enzmictec.2018.10.003

Yang, S-Y., Han, Y-H., Park, Y-L., Park, J-Y., No, S-Y., Jeong, D., Park, S., Park, H.Y., Kim, W., Seo, S-O., Yang, Y-H. (2020). Production of L-theanine Using *Escherichia coli* WHOLE-CELL OVEREXPRESSING γ-glutamylmethylamide synthetase with baker’s yeast. *J Microbiol Biotechnol*, 30 (5), 785. doi: 10.4014/jmb.1910.10044
